# Apoptotic and anti-proliferative effect of guanosine and guanosine derivatives in HuT-78 T lymphoma cells

**DOI:** 10.1007/s00210-020-01864-8

**Published:** 2020-04-20

**Authors:** Erich H. Schneider, Olga Hofmeister, Solveig Kälble, Roland Seifert

**Affiliations:** 1 Institute of Pharmacology, Medical School of Hannover, Carl-Neuberg-Str. 1, 30625 Hannover, Germany; 2 Institute of Molecular Toxicology and Pharmacology, Helmholtz Zentrum München-German Research Center for Environmental Health, Ingolstädter Landstrasse 1, 85764 Neuherberg, Germany

**Keywords:** Nucleoside transporters, Guanosine, Apoptosis, Proliferation, Leukemia, T-cells

## Abstract

The effects of 100 μM of 3′,5′-cGMP, cAMP, cCMP, and cUMP as well as of the corresponding membrane-permeant acetoxymethyl esters on anti-CD3-antibody (OKT3)-induced IL-2 production of HuT-78 cutaneous T cell lymphoma (Sézary lymphoma) cells were analyzed. Only 3′,5′-cGMP significantly reduced IL-2 production. Flow cytometric analysis of apoptotic (propidium iodide/annexin V staining) and anti-proliferative (CFSE staining) effects revealed that 3′,5′-cGMP concentrations > 50 μM strongly inhibited proliferation and promoted apoptosis of HuT-78 cells (cultured in the presence of αCD3 antibody). Similar effects were observed for the positional isomer 2′,3′-cGMP and for 2′,-GMP, 3′-GMP, 5′-GMP, and guanosine. By contrast, guanosine and guanosine-derived nucleotides had no cytotoxic effect on peripheral blood mononuclear cells (PBMCs) or acute lymphocytic leukemia (ALL) xenograft cells. The anti-proliferative and apoptotic effects of guanosine and guanosine-derived compounds on HuT-78 cells were completely eliminated by the nucleoside transport inhibitor NBMPR (S-(4-Nitrobenzyl)-6-thioinosine). By contrast, the ecto-phosphodiesterase inhibitor DPSPX (1,3-dipropyl-8-sulfophenylxanthine) and the CD73 ecto-5′-nucleotidase inhibitor AMP-CP (adenosine 5′-(α,β-methylene)diphosphate) were not protective. We hypothesize that HuT-78 cells metabolize guanosine-derived nucleotides to guanosine by yet unknown mechanisms. Guanosine then enters the cells by an NBMPR-sensitive nucleoside transporter and exerts cytotoxic effects. This transporter may be ENT1 because NBMPR counteracted guanosine cytotoxicity in HuT-78 cells with nanomolar efficacy (IC_50_ of 25–30 nM). Future studies should further clarify the mechanism of the observed effects and address the question, whether guanosine or guanosine-derived nucleotides may serve as adjuvants in the therapy of cancers that express appropriate nucleoside transporters and are sensitive to established nucleoside-derived cytostatic drugs.

**Electronic supplementary material:**

The online version of this article (10.1007/s00210-020-01864-8) contains supplementary material, which is available to authorized users.

## Introduction

Cyclic nucleotides (cNMPs), specifically cAMP and cGMP, are well-established second messengers. Although involvement in intracellular signaling processes is considered the main function of these cNMPs, an increasing body of scientific literature reports on first messenger effects of extracellular cAMP and cGMP. A “cAMP-adenosine pathway” has been repeatedly demonstrated for various cell types (Godinho et al. 2015; Jackson and Raghvendra 2004). 3′,5′-cAMP is exported into the extracellular space, followed by enzymatic degradation to adenosine, which in turn activates G protein-coupled receptors (Godinho et al. 2015, Jackson and Raghvendra 2004). A similar pathway seems to exist for 2′,3′-cAMP (Verrier et al. 2011) and for 2′,3′-cGMP (Jackson et al. 2019). In addition, several reports suggest that extracellular degradation products of 3′,5′-cGMP exert biological effects in the brain (Albrecht et al. 2013; Saute et al. 2006; Soares et al. 2004). Moreover, the pyrimidine nucleoside uridine activates adenosine receptors (Yilmaz et al. 2008). Thus, the non-canonical cyclic nucleotide cUMP, which is currently discussed as a potential second messenger (Seifert et al. 2015; Berrisch et al. 2017; Ostermeyer et al. 2018; Scharrenbroich et al. 2019), may as well be exported and degraded to biologically active products. Preliminary results suggest that first- and second messenger effects of cyclic nucleotides are highly dependent on the investigated cell type (Schneider et al. 2015).

Here we report on the effects of extra- and intracellular cNMPs and their extracellular degradation products on HuT-78 Sézary lymphoma cells in three different functional readouts, namely T cell receptor (TCR)-mediated IL-2 production, apoptosis, and proliferation. Unmodified cNMPs were used to address extracellular effects. Membrane-permeant 3′,5′-cNMP acetoxymethyl esters (cNMP-AMs) that release the unmodified 3′,5′-cNMPs after cleavage by intracellular esterases were used to investigate intracellular actions. IL-2 production of HuT-78 T cell lymphoma cells (induced with αCD3 antibody (OKT3) in the absence of αCD28) was significantly inhibited by 3′,5′-cGMP, while cGMP-AM was ineffective. Experiments addressing proliferation and apoptosis of HuT-78 cells (cultured in the presence of αCD3) revealed anti-proliferative and pro-apoptotic effects, not only of 3′,5′-cGMP but also of 2′,3′-cGMP, 2′-GMP, 3′-GMP, 5′-GMP, and guanosine. The effects of these guanosine-derived compounds depended on the activity of NBMPR (S-(4-Nitrobenzyl)-6-thioinosine)-sensitive nucleoside transporters. By contrast, substances not related to guanosine were ineffective. Our observations may be explained by the hypothesis that HuT-78 cells metabolize 3′,5′-cGMP, 2′,3′-cGMP, 2′-GMP, 3′-GMP, and 5′-GMP to guanosine, which enters the cells by an NBMPR-sensitive nucleoside transporter and exerts cytotoxic effects. Further studies are needed to investigate whether guanosine or guanosine-derived nucleotides could be used as adjuvants in the chemotherapy of lymphomas expressing appropriate nucleoside transporters.

## Materials and methods

### Buffers, reagents, and cell culture media

CFSE (5(6)-carboxyfluorescein diacetate N-succinimidyl ester; Cat.# 21888) and propidium iodide solution (Cat.# P4864) was provided by Sigma Aldrich (Taufkirchen, Germany). Annexin-V-APC (Cat.# AnxA100) was purchased from MabTag (Frisoythe, Germany). Mouse monoclonal (OKT3) anti-CD3 antibody (Cat.# SAB4700041) was from Sigma Aldrich (Taufkirchen, Germany). Pacific blue-labeled anti-human CD3 (Cat.# 300417) was obtained from Biolegend (London, UK). Anti-CD28 antibody (Cat.# SAB4700135–100 μg) was purchased from Sigma Aldrich (Taufkirchen, Germany). For detection of IL-2, either the Duoset ELISA (Cat.# DY202) from R&D Systems (Minneapolis, MN, USA) was used or the ELISA MAX Standard Set for human IL-2 (Cat.# 431801) from Biolegend (London, UK).

DPSPX (1,3-dipropyl-8-p-sulfophenylxanthine; Cat.# A022) and NBMPR (S-(4-nitrobenzyl)-6-thioinosine; Cat.# N2255) were obtained from Sigma Aldrich (Taufkirchen, Germany). 2′,3′-cGMP (Cat.# G025–50), 3′,5′-cGMP (Cat.# G001–100), 2′-GMP (Cat.# G022–10), 3′-GMP (Cat.# G021–10), and AMP-CP (adenosine-5′-(α,β-methylene)diphosphate, sodium salt; Cat.# A070) were purchased from Biolog (Bremen, Germany). 5′-GMP disodium salt (Cat.# G8377) and guanosine (Cat. # G6752) were provided by Sigma Aldrich (Taufkirchen, Germany).

RPMI 1640 medium (Cat.# R8758) and fetal calf serum (Cat.# F7524) were purchased from Sigma Aldrich (Taufkirchen, Germany). L-Glutamine with penicillin/streptomycin (Gibco # 10378–016), MEM non-essential amino acids (100x) (Gibco # 11140–035), sodium pyruvate 100 mM (100x) (Gibco # 11360–039), and AIMV medium (Gibco # 12055–091) were obtained from Thermo Fisher Scientific (Waltham, USA). Trypsin/EDTA 0.25% (Cat.# T4049) as well as PBS (Dulbecco’s phosphate buffered saline, 10 x, sterile) were provided by Sigma Aldrich (Taufkirchen, Germany). Biocoll Separating Solution (Cat.# L6115) for isolation of PBMCs was obtained from Merck, Berlin, Germany.

### Cell culture

HuT-78 cutaneous T lymphoma cells (Sézary lymphoma) were obtained from LGC Standard GmbH (Wesel, Germany) and maintained in RPMI 1640 medium supplemented with 10% (v/v) of fetal bovine serum and 2 mM of L-glutamine as well as 100 U/ml penicillin and 0.1 mg/ml streptomycin. Moreover, the medium was supplemented with 1% (v/v) of MEM non-essential amino acids (100 x) and 1 mM of sodium pyruvate (addition of 100 mM stock solution). The cells were cultured at 37 °C in the presence of 5% of CO_2_. Medium was renewed three times a week by diluting the cells to yield a cell density of 1 × 10^5^ cells on Mondays and Wednesdays and 5 × 10^4^ cells on Fridays.

The acute lymphatic leukemia (ALL) xenografts were obtained from Dr. Beat Bornhauser (Department of Pediatric Oncology, Children’s Research Centre, University Children’s Hospital Zürich, Zürich, Switzerland). The ALL cells were thawed and seeded in AIMV medium at a density of 2.5 × 10^5^ cells/ml in 500 μl per well and on a layer of mesenchymal stem cells (MSC). The MSC cells were cultivated in RPMI 1640 medium containing 10% (v/v) fetal bovine serum, 2 mM L-Glutamine, 100 U/ml penicillin, and 0.1 mg/ml streptomycin. MSC cells were split 1:5 twice a week (detachment by 0.25% Trypsin/EDTA).

Peripheral blood monocytic cells (PBMCs) were obtained from healthy human blood donors. The monocytes were separated by centrifugation with Biocoll separating solution and then seeded on anti-CD3-antibody-coated plates and in RPMI 1640 medium supplemented with 10% (v/v) of fetal bovine serum, 2 mM of L-glutamine, 100 U/ml penicillin, 0.1 mg/ml streptomycin, and 4 μg/ml anti-CD28 antibody.

### IL-2 ELISA

96-well plates were coated for 24 h with 50 μl of PBS containing 2 μg/ml of αCD3 antibody (OKT3). On the next day, the plates were washed twice with 1× PBS and then loaded with 50 μl of cell suspension/well (100,000 HuT-78 cells or 1.75 × 10^5^ PBMCs per well) plus 50 μl media with the corresponding stimuli. After 24 h of incubation, the plate was centrifuged for 10 min at 500×*g*. After that, supernatant was removed and used for the ELISA experiment. ELISAs were performed according to the manufacturer’s instructions (Duoset, Cat. # DY 202, from R&D Systems or ELISA MAX Standard Set, Cat. # 431801, from Biolegend).

### Apoptosis assay with HuT-78 cells, ALL-cells, and PBMCs

HuT-78 cells (1 × 10^5^ cells/ml) were treated for 72 h (37 °C, 5% CO_2_) in αCD3-antibody-coated 24-well plates with cNMPs, NMPs, or nucleosides in the presence or absence of inhibitors (AMP-CP, DPSPX, NBMPR). To counteract potential degradation in case of incubation with AMP-CP or DPSPX, 100 μM of these inhibitors were not only added at the beginning of the incubation time but also after 24 h and 48 h. Thus, after 72 h, the samples contained up to 300 μM of AMP-CP or DPSPX. On the day of the measurement, the cells were centrifuged (300×*g*, 4 min, ambient temperature), suspended in 100 μl of binding buffer (10 mM HEPES, 140 mM NaCl, 2.5 mM CaCl_2_, pH 7.4) and then incubated with annexin-V-APC for 30 min in the dark at ambient temperature. After that, each sample was diluted by addition of 200 μl of binding buffer. Propidium iodide (PI) solution (final concentration 625 ng/ml) was added immediately prior to flow cytometric quantitation of apoptosis, which was performed using a MACSQuant Analyzer (Miltenyi Biotech, Bergisch Gladbach, Germany). Apoptosis was analyzed by generating an annexin-APC/PI dot plot (APC/PI), which allows the discrimination of the following populations: lower left (LL) quadrant (annexin-V-APC- and PI-negative): viable cells; lower right (LR) quadrant (annexin-V-APC-positive, PI-negative): early apoptosis; upper right (UR) quadrant (positive for both annexin-V-APC and PI): late apoptosis and upper left (UL) quadrant (positive for PI, negative for annexin-V-APC: necrosis. The data were analyzed with the MACSQuantify software. The degree of apoptosis was defined as percentage of total cells in the LR and UR quadrant (=% apoptic cells).

In case of ALL cells, MSC feeder cells were seeded on Fridays on a 24-well plate (pre-coated for 24 h with 2 μg/ml of α-CD3 antibody) at a density of 1 × 10^4^ cells/ml (1 ml per well). Three days later, 500 μl of medium were removed and the ALL cells were seeded on top of the MSC layer. After incubation with cNMPs, NMPs, nucleosides, and/or NBMPR, the cells were separated from the MSC layer by rinsing with a pipet tip. This led to detachment of the ALL cells, while the MSC cells remained adherent. The ALL cells were centrifuged for 5 min at 300×*g* and then suspended in 100 μl of binding buffer. After that, the cells were incubated with annexin-V-APC for 15 min, followed by an additional incubation step with Pacific Blue anti-human CD3 antibody for 15 min in the dark at ambient temperature. Cells were washed at 300×*g* for 5 min and then diluted in 300 μl of binding buffer. Apoptosis was determined as described above after addition of PI.

PBMCs were seeded at a density of 1.75 × 10^5^ cells per ml in 1 ml per well on an anti-CD3 antibody-coated 24-well plate with medium containing anti-CD28 antibody. Flow cytometric analysis of apoptosis was performed using the Annexin V/PI method as described above. Similar to the procedure used for the ALL cells, the PBMCs were also stained with Pacific blue-labeled anti-human CD3, and only the cells with the highest fluorescence were gated for flow cytometric analysis of apoptosis.

### HuT-78 cell proliferation assay

An appropriate number of cells was centrifuged (300×*g*, 4 min) and resuspended in a minimum of 1 ml of PBS with 0.1% of bovine serum albumin (BSA), yielding a density of 6.7 × 10^6^ cells/ml. After that, an appropriate volume of a 250 μM of CFSE (carboxyfluorescein succinimidyl ester) stock solution was added to yield a final CFSE concentration of 250 nM. The samples were incubated for 10 min at 37 °C, followed by centrifugation (300×*g*, 4 min) and resuspension in medium to yield a density of 1 × 10^5^ cells/ml. This suspension was seeded in an αCD3-antibody-coated 24-well plate (1 ml/well) and incubated for 72 h (37 °C, 5% CO_2_) with cNMPs, NMPs, or nucleosides in the presence or absence of inhibitors (AMP-CP, DPSPX, NBMPR). To counteract potential degradation in case of incubation with AMP-CP or DPSPX, 100 μM of these inhibitors were not only added at the beginning of the incubation time but also after 24 h and 48 h. Thus, after 72 h, the samples contained up to 300 μM of AMP-CP or DPSPX. On the day of the measurement, the content of each well (1 ml) was centrifuged (300×*g*, 4 min) and resuspended in 1 ml of FACS buffer. Proliferation was analyzed by flow cytometric quantitation of residual cell-bound CFSE fluorescence using a MACSQuant Analyzer (Miltenyi Biotech, Bergisch Gladbach, Germany).

### Statistics and data analysis

Statistical analysis of the data and generation of diagrams was performed with GraphPad Prism 6.07 (GraphPad Software Inc., San Diego, CA, USA). The statistical tests are detailed in the figure legends. All data are provided as means ± SD.

## Results

### Effect of cyclic nucleotides on anti-CD3 antibody-induced IL-2 production of HuT-78 lymphoma cells

**Fig. 1:**
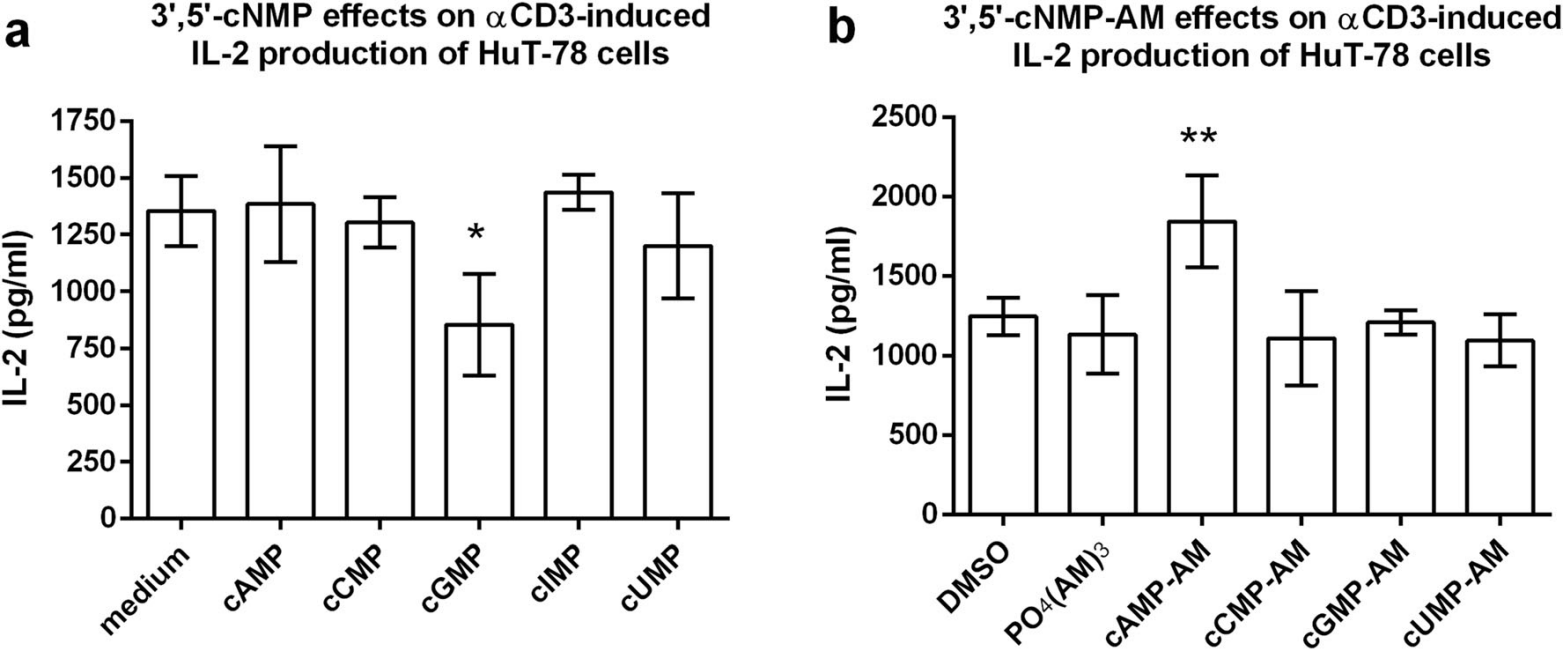
Effect of unmodified cNMPs and of membrane-permeant cNMP-AM esters on αCD3-antibody-induced IL-2 production of HuT-78 lymphoma cells. Cells were incubated for 24 h in αCD3-antibody-coated plates with 100 μM of cNMPs (**a**) or 100 μM of cNMP-AMs (**b**) The “DMSO” and the “PO_4_(AM)_3_” bars represent controls for the DMSO content of the cNMP-AM samples and for the intracellular hydrolysis products of the AM esters. Data shown are means ± SD from *n* = 3 independent experiments. Statistics: one-way ANOVA and Dunnet’s multiple comparison test with medium (**a**) or PO_4_(AM)_3_ (**b**) as control columns. Asterisks indicate significance level: * = *p* < 0.05; ** = *p* < 0.01

The effect of 100 μM of the purine cNMPs 3′,5′-cAMP, 3′,5′-cGMP, and 3′,5′-cIMP as well as of the pyrimidine cNMPs 3′,5′-cUMP and 3′,5′-cCMP on αCD3-antibody-induced IL-2 production of HuT-78 lymphoma cells was analyzed by ELISA. Additionally, the corresponding membrane-permeable acetoxymethyl ester analogues (cNMP-AMs) were tested. For unknown reasons, anti-CD3 antibody-induced IL-2 production of HuT-78 cells showed high inter-experimental variability, although the conditions were kept essentially the same in all experiments. Thus, the experiments with almost no stimulation of IL2 production were excluded from analysis. The data from the experiments with high IL2 production, however, clearly differentiate between extra- and intracellular effects of the purine cNMPs. IL-2 release was not affected by unmodified cAMP (Fig. 1a) but significantly enhanced by cAMP-AM (Fig. 1b). By contrast, IL-2 production was significantly reduced by unmodified cGMP (Fig. 1a) but was not modulated by the structurally closely related cIMP (Fig. 1a) and also not by cGMP-AM (Fig. 1b). The unmodified pyrimidine cyclic nucleotides cCMP and cUMP (Fig. 1a) as well as their AM derivatives (Fig. 1b) were ineffective.

### Anti-proliferative and apoptotic effects of 3′,5′- and 2′,3′-cGMP and their potential metabolic products on HuT-78 lymphoma cells

**Fig. 2:**
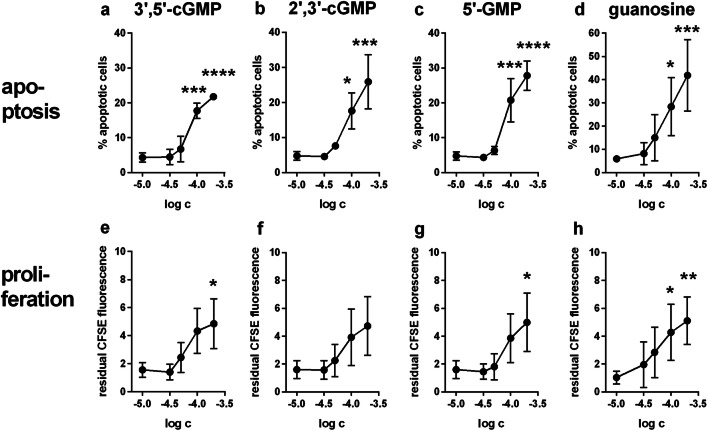
Dose-dependent effect of 3′,5′-cGMP, 2′,3′-cGMP, 5′-GMP and guanosine on apoptosis and proliferation of HuT-78 lymphoma cells in the presence of αCD3-antibody. Apoptosis (**a**–**d**) and proliferation (**e**–**h**) were determined by flow cytometry after 72 h of incubation with increasing concentrations of 3′,5′-cGMP (**a**, **e**), 2′,3′-cGMP (**b**, **f**), 5′-GMP (**c**, **g**), and guanosine (**d**, **h**). Apoptosis was determined by combined staining with APC-labeled annexin V and propidium iodide; the anti-proliferative effect of cNMPs was determined via measuring the residual CFSE fluorescence at the end of the incubation time. Please note that increased CFSE fluorescence means reduced proliferation (inverted relationship). Data are means ± SD from *n* = 3 (2′,3′-cGMP, 3′,5′-cGMP, and 5′-GMP) or *n* = 4 (guanosine) independent experiments. In all graphs, the data point depicted at log − 5.0 represents the medium control and not a “real” 10 μM concentration. Statistics: one-way ANOVA and Dunnet’s multiple comparison test with medium control (data point at log − 5.0) as control value. Asterisks indicate significance level: * = *p* < 0.05; **= *p* < 0.01; *** = *p* < 0.001 and **** = *p* < 0.0001

To investigate whether the inhibitory effect of extracellular 3′,5′-cGMP on IL-2 release is associated with a reduction of HuT-78 cell proliferation and/or viability, the influence of 3′,5′-cGMP on proliferation and apoptosis of HuT-78 cells was analyzed in a concentration range from 10 to 200 μM. The experiments were performed in the presence of αCD3 antibody and with an incubation time of 72 h. At baseline, only about 5% of the HuT-78 cells were apoptotic (5.1 ± 1.8%; *n* = 8 independent experiments; mean ± SD). However, this proportion was significantly raised to 17.7 ± 2.2% and to 21.8 ± 0.6% in the presence of 100 μM and 200 μM of 3′,5′-cGMP, respectively (Fig. 2a). Representative flow cytometric raw data (scattergrams) are depicted in the supplemental information to illustrate the baseline signal (Suppl. Fig. 1a) and the apoptosis in the presence of 100 μM of 3′,5′-cGMP (Suppl. Fig. 1b). Interestingly, a similar pro-apoptotic effect (significant at 100 μM and 200 μM) was also found with the structurally closely related positional isomer 2′,3′-cGMP (Fig. 2b).

**Fig. 3:**
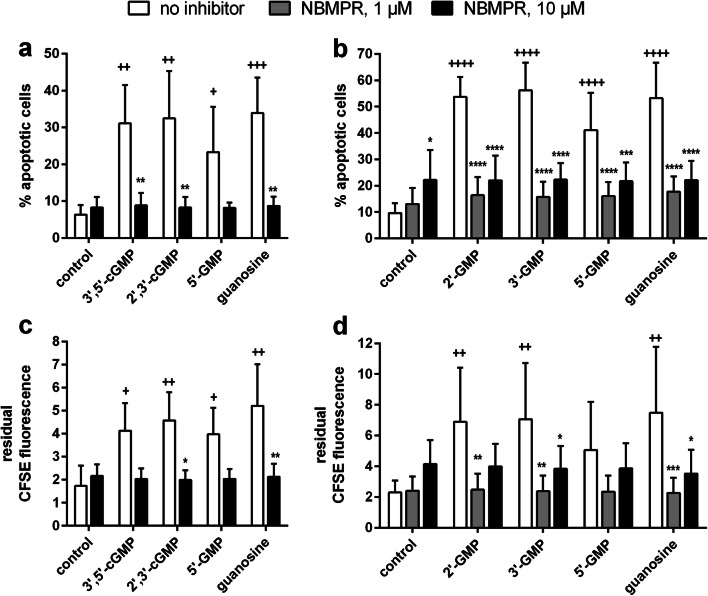
Effect of the nucleoside transporter inhibitor NBMPR (1 μM and/or 10 μM) on apoptosis (**a**, **b**) and proliferation (**c**, **d**) of αCD3-antibody-stimulated HuT-78 cells treated with various guanosine-derived compounds (100 μM). Two independent sets of experiments were conducted. Initially, the experiments included only 10 μM of NBMPR (**a**, **c**). Later, 1 μM of NBMPR was additionally included (**b**, **d**). For statistical reasons, the two sets were separately depicted and analyzed (two-way ANOVA not possible with partially missing groups). HuT 78 cells were incubated with the test compounds for 72 h in the presence of anti-CD3 antibody (OKT3). The samples contained no inhibitor (open bars) or 1 μM of NBMPR (gray bars) or 10 μM of NBMPR (black bars). Apoptosis (**a**, **b**) and proliferation (**c**, **d**) were determined by flow cytometry after 72 h of incubation (apoptosis: staining with APC-labeled annexin V and propidium iodide; proliferation: residual CFSE fluorescence at the end of the incubation time, higher residual CFSE fluorescence means less proliferation). Statistical results: Fig. 3a, c: Two-way ANOVA and Dunnet’s multiple comparison test for comparison of guanosine nucleotide effects vs. control column; two-way ANOVA with Sidak’s multiple comparisons test for comparison of inhibitor-containing samples vs. corresponding inhibitor-free controls. Figure 3b, d: two-way ANOVA and Dunnet’s multiple comparison test for comparison of guanosine nucleotide effects vs. control column and for comparison of inhibitor-containing samples vs. corresponding inhibitor-free controls. One, two, three, and four symbols designate *p* < 0.05, *p* < 0.01, *p* < 0.001, and *p* < 0.0001, respectively. Significant differences between NBMPR-containing samples and the corresponding inhibitor-free samples are indicated by “*”. Significant differences in comparison to the corresponding control columns are indicated by “+”. Number of independent experiments: **a**, **c**: *n* = 3 for both apoptosis and proliferation; **b**, **d**: *n* = 6 (apoptosis) and *n* = 5 (proliferation)

We hypothesized that the effects of 3′,5′-cGMP and 2′,3′-cGMP on HuT-78 cell apoptosis could be due to common metabolic products formed by enzymes on the cell surface. To test this hypothesis, the effects of 5′-GMP (potential 3′,5′-cGMP hydrolysis product) and guanosine (possibly formed by degradation of 3′,5′-cGMP and/or 2′,3′-cGMP) were investigated in apoptosis assays. In fact, both 5′-GMP (Fig. 2c) and guanosine (Fig. 2d) increased apoptosis of HuT-78 cells. This effect reached significance at concentrations of 100 μM and 200 μM. In addition, in later experiments, we also demonstrated that 100 μM of 2′-GMP (potential metabolite of 2′,3′-cGMP) and 3′-GMP (potential metabolite of 2′,3′- and 3′,5′-cGMP) exerted a highly significant apoptotic effect after 72 h of incubation (Fig. 3b**)**.

Since pro-apoptotic action might be associated with an inhibition of proliferation, we also investigated the effect of the aforementioned guanosine-derived substances on HuT-78 cell proliferation in the CFSE assay. Residual CFSE fluorescence after 72 h of incubation was considerably increased by 100 μM as well as 200 μM of 3′,5′-cGMP, 2′,3′-cGMP, 5′-GMP, and guanosine (Fig. 2e–h), indicating a strong anti-proliferative effect. Due to the large inter-experimental variability, however, the anti-proliferative effect only reached significance in case of 3′,5′-cGMP (at 200 μM), of 5′-GMP (at 200 μM) and of guanosine (at 100 μM and 200 μM) (Fig. 2e–h). In general, the apoptotic and anti-proliferative effects of the investigated guanosine-related compounds started to appear at a concentration of 50 μM, followed by a steep increase of activity between 50 and 100 μM (Fig. 2a–h). A similarly pronounced anti-proliferative effect was observed in later experiments performed under the same conditions with 100 μM of 2′-GMP (potential metabolite of 2′,3′-cGMP) and of 3′-GMP (potential metabolite of 2′,3′- and 3′,5′-cGMP) (Fig. 3d**)**. Interestingly, as shown in Suppl. Fig. 2, 100 μM of the membrane-permeable cGMP-AM also exerted a strong apoptotic (Suppl. Fig. 2a) and anti-proliferative (Suppl. Fig. 2b) effect on HuT-78 cells, which is in contrast to its missing or only weak effect on αCD3-induced IL-2 release in Fig. 1 or in Suppl. Fig. 3.

### Inhibition of anti-CD3 antibody-induced IL-2 production by products of 2′,3′-cGMP and/or 3′,5′-cGMP metabolism

The inhibitory effect of extracellular 3′,5′-cGMP on IL-2 release of HuT-78 cells is associated with reduced HuT-78 cell proliferation and viability. In addition, later experiments have shown that the positional isomer 2′,3′-cGMP as well as the 2′,3′-cGMP/3′,5′-cGMP metabolites 2′-GMP, 3′-GMP, 5′-GMP, and guanosine also exert apoptotic and anti-proliferative effects. This prompted us to investigate whether these metabolites also inhibit IL-2 release. Indeed, ELISA experiments demonstrated that IL2 release of HuT-78 cells (in the presence of αCD3)-free samples is effectively inhibited by 2′-GMP, 3′-GMP, 5′-GMP, and guanosine (Suppl. Fig. 3).

However, the effects did not reach significance, and the absolute amount of IL-2 released was much lower than in previous experiments (compare Fig. 1 and Suppl. Fig. 3). The “w/o α-CD3” control column in Suppl. Fig. 3 indicates that the HuT-78 cells showed already α-CD3-independent baseline IL-2 production in these experiments, and IL-2 release was not further enhanced by α-CD3 antibody. By contrast, in our previous experiments (Fig. 1), the IL-2 production by HuT-78 cells was so effectively increased by α-CD3 that the control column for α-CD3 was omitted because it was almost zero and did not provide meaningful information. We have no explanation for the large and unpredictable discrepancies in IL-2 release from HuT-78 cells. By contrast, the apoptosis and proliferation measurements showed considerably better inter-experimental reproducibility. For this reason, we focused on apoptosis and proliferation assays for the rest of the project.

### Role of equilibrative nucleoside transporters in mediating cytotoxicity of guanosine-derived compounds

We hypothesized that both 3′,5′-cGMP and 2′,3′-cGMP are first converted to mononucleotides by an ecto-phosphodiesterase on the cell surface, followed by the formation of guanosine by ectonucleotidases. This would mean that guanosine is the common anti-proliferative and pro-apoptotic metabolic end-product of 3′,5′-cGMP, 2′,3′-cGMP, 5′-GMP, 3′-GMP, and 2′-GMP. Guanosine may then enter HuT-78 cells via a nucleoside transporter and unfold its cytotoxic effects from inside the cell.

To test this hypothesis, HuT-78 cells were incubated with guanosine and guanosine nucleotides in the presence and absence of S-(4-Nitrobenzyl)-6-thioinosine (NBMPR), which preferentially inhibits the human equilibrative nucleoside transporter hENT1. According to the literature, the IC_50_ value for nucleoside transport inhibition by NBMPR is 0.4 nM and 2.8 μM for hENT1 and hENT2, respectively (Ward et al. 2000). Thus, we used a concentration of 10 μM of NBMPR, which should be sufficient to completely inhibit guanosine uptake by hENT1 and which should only partially block hENT2. Again, 100 μM of guanosine, 5′-GMP, 2′,3′-cGMP, or 3′,5′-cGMP significantly promoted apoptosis (Fig. 3a) and inhibited proliferation (Fig. 3c) (two-way ANOVA and Dunnet’s multiple comparison test, comparisons with corresponding control column). The hENT1 inhibitor NBMPR was clearly protective and inhibited the pro-apoptotic and anti-proliferative actions of the tested guanosine-derived compounds (Fig. 3a, c). The protective NBMPR effect reached significance for 2′,3′-cGMP, 3′,5′-cGMP, and guanosine in the apoptosis experiments (Fig. 3a) and for 2′,3′-cGMP and guanosine in the proliferation assay (Fig. 3c) (two-way ANOVA with Sidak’s multiple comparisons test for comparison of inhibitor-containing samples with corresponding inhibitor-free controls). Later in the project, we performed a second set of experiments that included not only 10 μM of NBMPR but also the lower concentration of 1 μM. Even at a concentration of only 1 μM, NBMPR protected HuT-78 cells from the cytotoxic effects of 100 μM of the 2′,3′-cGMP and/or 3′,5′-cGMP metabolites 2′-GMP, 3′-GMP, 5′-GMP, and guanosine. This is illustrated for apoptosis in Fig. 3b and for proliferation in Fig. 3d. Interestingly, Fig. 3b, d show that NBMPR has already a maximum protective effect at a concentration of 1 μM. By contrast, 10 μM of NBMPR seem to be slightly cytotoxic, as indicated by a weak apoptotic and anti-proliferative effect (Fig. 3b, d).

Moreover, NBMPR counteracted the inhibitory effect of 2′-GMP, 3′-GMP, 5′-GMP, and guanosine on IL-2 release, although the effects did not reach significance (ELISA data in Suppl. Fig. 3). Suppl. Fig. 3 again indicates that 1 μM of NBMPR is already sufficient to cause the maximum protective effect in the IL-2 assay.

### Determination of the IC_50_ value of NBMPR

The results reported so far suggest that 2′,3′-cGMP and 3′,5′-cGMP are metabolized to the end-product guanosine, which is taken up into the cell by an NBMPR-sensitive nucleoside transporter and then exerts apoptotic and anti-proliferative effects. The two most important nucleoside transporters for guanosine, ENT1 and ENT2, show largely different IC_50_ values for NBMPR (see Table 1: 0.4 nM for ENT1 and 2.8 μM for ENT2). A third, yet undefined transporter is responsible for the *csg* guanosine transport process and is inhibited by NBMPR with a K_i_ value of 0.7 nM.

**Table 1:** Important transport processes for nucleosides and nucleoside analogues

Process	Protein	Expression	Examples for physiological substrates (K_m_)	Examples for nonphysiological substrates (K_m_)	NBMPR sensitive? (IC_50_)
Equilibrative, bidirectional facilitators (ENT transporter family: SLC29)					
*es*	hENT1	ubiquitous	adenosine (50 μM) guanosine (140 μM) inosine (200 μM) uridine (480 μM) thymidine (240 μM) cytidine (680 μM)	gemcitabine (160 μM) cytarabine fludarabine cladribine	yes (0.4 nM)
*ei*	hENT2	ubiquitous, high abundance in skeletal muscle	adenosine (140 μM) guanosine (2700 μM) inosine (50 μM) uridine (270 μM) thymidine (620 μM) cytidine (5210 μM)	gemcitabine (740 μM) cladribine cytarabine fludarabine	no (2.8 μM)
*–*	hENT3	ubiquitous, e.g. placenta, mainly intracellular, optimum activity at pH 5.5	adenosine (1900 μM) uridine (2000 μM)	e.g. gemcitabine	no
*–*	hENT4 (PMAT)	ubiquitous	adenosine (780 μM), organic cations including serotonin (1900 μM)	1-methyl-4-phenylpyridinium (MPP^+^, neurotoxin)	no
Concentrative, inwardly directed sodium/nucleoside cotransporters (CNT transporter family: SLC 28)					
*cit*	hCNT1	e.g. epithelial tissues of small intestine, kidney and liver	adenosine, pyrimidine nucleosides	zidovudine, lamivudine, gemcitabine (17 μM), cytarabine, 5′-DFUR (209 μM)	no
*cif*	hCNT2	numerous tissues, e.g. kidney, liver, heart, brain, placenta, pancreas	purine nucleosides, uridine	didanosine, ribavirin	no
*cib*	hCNT3	e.g. pancreas, trachea, bone marrow, mammary gland	purine and pyrimidine nucleosides	gemcitabine, fludarabine, cladribine	no
*csg*	???	NB4 promyelocytic leukemia cells, L1210 murine acute lymphocytic leukemia cells	guanosine	???	yes (0.7 nM)

The information in this table comes from the following publications: (Flanagan and Meckling-Gill 1997; Ward et al. 2000; Young et al. 2008; Pastor-Anglada et al. 2004; Gray et al. 2004; Govindarajan et al. 2009). Please note that this table is not exhaustive and does not completely cover the literature on substrate selectivity of nucleoside transporters. Abbreviations: PMAT = Plasma membrane monoamine transporter; 5'-DFUR = 5'-Deoxy-5-fluorouridine

**Fig. 4:**
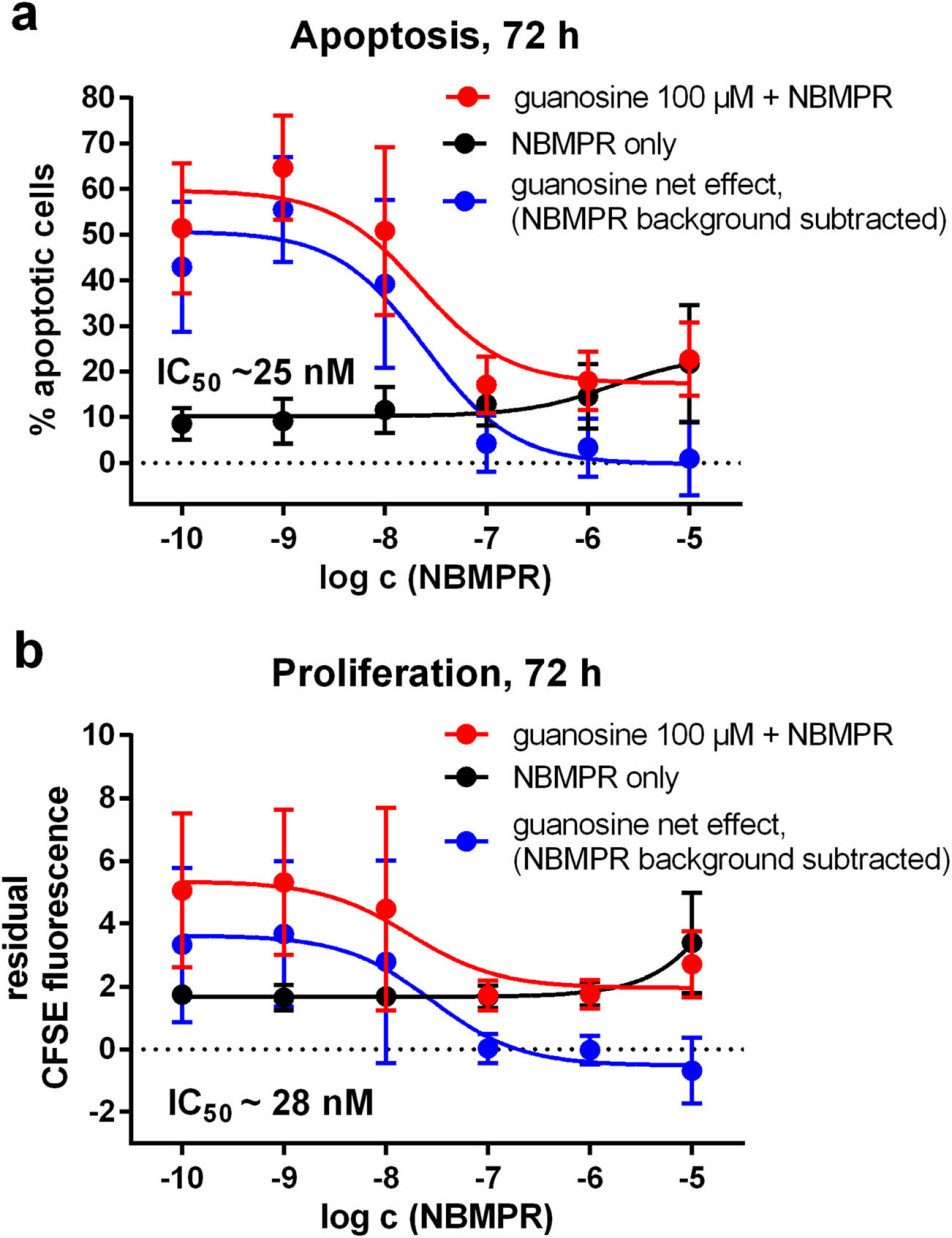
Effect of increasing concentrations of NBMPR on apoptosis (**a**) and proliferation (**b**) of HuT-78 cells in the presence (red curve) and in the absence (black curve) of 100 μM of guanosine. The blue curve represents the difference between red and black curve, i.e. the net effect of guanosine after subtraction of the “NBMPR background”. Apoptosis (**a**) and proliferation (**b**) were determined by flow cytometry after 72 h of incubation (apoptosis: combined staining with APC-labeled annexin V and propidium iodide; proliferation: quantitation of residual CFSE fluorescence at the end of the incubation time, higher residual CFSE fluorescence means less proliferation). Data are means ± SD from *n* = 5 (apoptosis) and *n* = 3 (proliferation) independent experiments

To learn more about the identity of the transporter responsible for the observed guanosine effects in HuT-78 cells, we recorded concentration-effect curves for apoptosis (Fig. 4a) and proliferation (Fig. 4b) with increasing concentrations of NBMPR in the presence of 100 μM of guanosine (red curves in Fig. 4). Since NBMPR exerts an apoptotic and anti-proliferative effect by itself at higher concentrations (cf. data shown in the preceding section), we additionally determined a concentration-effect curve in the absence of guanosine (black curves in Fig. 4). This background effect of NBMPR alone was subtracted from the guanosine + NBMPR data, yielding the net effect of guanosine in the presence of increasing NBMPR concentrations (blue curves in Fig. 4). The IC_50_ value of NBMPR was ~ 25 nM in the apoptosis assays and ~ 28 nM in the proliferation experiments.

### Role of ecto-phosphodiesterase and ecto-5′-nucleotidase in mediating cytotoxicity of guanosine nucleotides

**Fig. 5:**
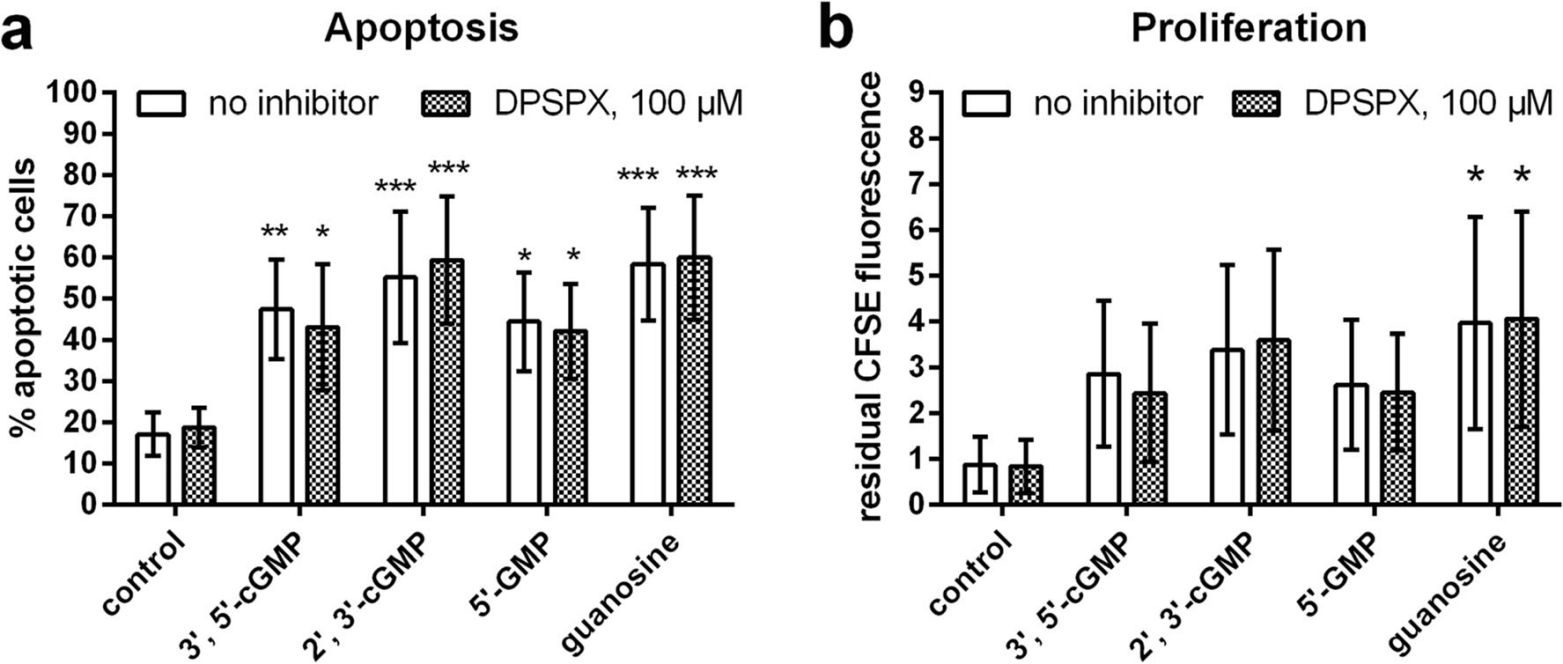
Effect of the ecto-phosphodiesterase inhibitor DPSPX on the pro-apoptotic (**a**) and anti-proliferative (**b**) actions of 100 μM of 3′,5′-cGMP, 2′,3′-cGMP, 5′-GMP, and guanosine on αCD3-antibody-stimulated HuT-78 lymphoma cells. Cells were incubated with guanosine-related compounds in the absence (open bars) or presence (filled bars) of the ecto-phosphodiesterase inhibitor DPSPX. 100 μM of fresh inhibitor were added at *t* = 0 h, 24 h, and 48 h to compensate for potential hydrolysis (maximum possible concentration at 72 h: 300 μM). Apoptosis (**a**) and proliferation (**b**) were determined by flow cytometry after 72 h of incubation. Apoptosis was determined by combined staining with APC-labeled annexin V and propidium iodide; the antiproliferative effect of cNMPs was determined via measuring the residual CFSE fluorescence at the end of the incubation time. Higher residual CFSE fluorescence means less proliferation. Statistics: asterisks indicate significant effect of treatment with guanosine-derived compounds (two-way ANOVA and Dunnet’s multiple comparison test, comparisons with corresponding control column). Data are means ± SD from *n* = 4 independent experiments

After having shown that NBMPR-sensitive nucleoside transport is the common mechanism for the cytotoxic effects of guanosine and guanosine-derived nucleotides, we addressed the steps that may lead to guanosine formation from the corresponding cyclic nucleotides and mononucleotides. First, we investigated the effect of DPSPX (1,3-dipropyl-8-sulfophenylxanthine), which is not only a well-known adenosine receptor antagonist but also an inhibitor of ecto-phosphodiesterases. Cells were incubated with 100 μM of DPSPX during the first 24 h. After 24 h and 48 h, 100 μM of fresh DPSPX were added to compensate for potential hydrolytic inactivation. Thus, in the absence of DPSPX hydrolysis, the DPSPX concentration in the samples would reach 300 μM after 72 h. However, DPSPX did not inhibit the pro-apoptotic (Fig. 5a) and anti-proliferative (Fig. 5b) effects of 2′,3′-cGMP, 3′,5′-cGMP, 5′-GMP, and guanosine.

**Fig. 6:**
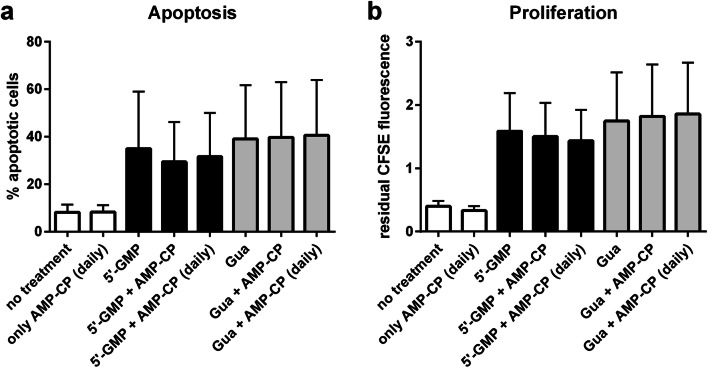
Effect of the 5′-ectonucleotidase (CD73) inhibitor AMP-CP on the pro-apoptotic (**a**) and anti-proliferative (**b**) actions of 100 μM of 5′-GMP or guanosine (HuT-78 lymphoma cells in the presence of αCD3 antibody). Cells were incubated with guanosine-derived compounds (black bars: 5′-GMP; gray bars: guanosine) in the absence or presence of 100 μM of the ecto-5'-nucleotidase inhibitor AMP-CP as designated in the x-axis labeling. The inhibitor was either added only once in the beginning at *t* = 0 h, or it was added freshly every day (at *t* = 0 h, 24 h, and 48 h; indicated as “AMP-CP (daily)” in the labeling of the bars). In both cases, the total incubation time lasted 72 h. Apoptosis (**a**) and proliferation (**b**) were determined by flow cytometry. Apoptosis was determined by combined staining with APC-labeled annexin V and propidium iodide; the anti-proliferative effect was determined via measuring the residual CFSE fluorescence at the end of the incubation time. Higher residual CFSE fluorescence means lower proliferation. Data are means ± SD from *n* = 2 independent experiments

Second, we analyzed whether AMP-CP (adenosine 5′-(α,β-methylene)diphosphate), an inhibitor of the ecto-5'-nucleotidase CD73, was able to modulate the effects of 5′-GMP on HuT-78 cell apoptosis and proliferation. Since guanosine would not require CD73 for its activation, it was selected as a negative control. Even after 72 h of incubation with 100 μM of AMP-CP, no alteration of the pro-apoptotic (Fig. 6a) or anti-proliferative (Fig. 6b) effects of 5-GMP (black bars in Fig. 6a, b) and guanosine (gray bars in Fig. 6a, b) was observed. To compensate for potential hydrolysis of AMP-CP, other samples were run in parallel, where 100 μM of fresh AMP-CP was added after 24 h and 48 h (similar as described above for DPSPX; bars labeled with “AMP-CP (daily)” in Fig. 6a, b). However, no effect of AMP-CP was observed in these samples either.

### Effects of 3′,5′-cIMP, 3′,5′-cAMP, and adenosine as well as of 2′,3′- and 3′,5′-cyclic pyrimidine nucleotides on HuT-78 cell apoptosis and proliferation

**Fig. 7:**
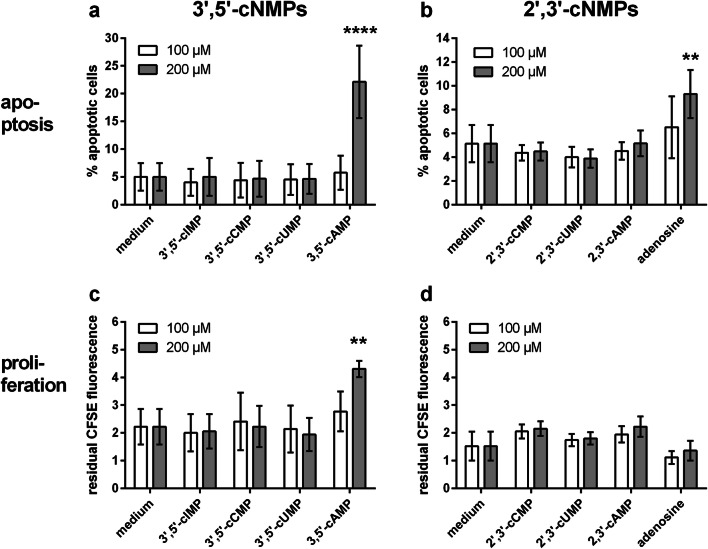
Effect of unmodified non-guanosine-related 3′,5′- and 2′,3′-cNMPs and adenosine on apoptosis and proliferation of αCD3-antibody-stimulated HuT-78 lymphoma cells. Cells were incubated with 100 μM (open bars) or 200 μM (filled bars) of 3′,5′-cNMPs (**a**, **c**) or 2′,3′-cNMPs (**b**, **d**). Apoptosis (**a**, **b**) and proliferation (**c**, **d**) were determined by flow cytometry after 72 h of incubation. Apoptosis was determined by combined staining with APC-labeled annexin V and propidium iodide; the anti-proliferative effect of cNMPs was determined via measuring the residual CFSE fluorescence at the end of the incubation time. Higher residual CFSE fluorescence means less proliferation. Data are means ± SD from *n* = 3 (2′,3′- and 3′,5′-cNMPs) or *n* = 2 (adenosine) independent experiments. Statistics: two-way ANOVA and Dunnet’s multiple comparison test (related to medium as control). Separate comparisons were performed for the 100 μM and the 200 μM column. Asterisks indicate significance level: * = p < 0.05; ** = p < 0.01 and **** = p < 0.0001

As shown in Fig. 1, unlike 3′,5′-cGMP, the structurally closely related cyclic nucleotide 3′,5′-cIMP had no influence on αCD3-antibody-induced IL2-production. Therefore, we investigated if this also pertains to apoptosis and proliferation. In fact, neither 100 μM nor 200 μM of 3′,5′-cIMP altered apoptosis (Fig. 7a) or proliferation (Fig. 7c) of HuT-78 cells. The other purine cyclic nucleotide, 3′,5′-cAMP, was also ineffective at 100 μM in both readouts (Fig. 7a, c), confirming the results from the IL2 ELISA experiments. Only at a concentration of 200 μM, 3′,5′-cAMP exerted a highly significant apoptotic and anti-proliferative effect (apoptosis: *p* < 0.0001; proliferation: *p* < 0.01; compared with medium control; two-way ANOVA with Dunnet’s multiple comparison test; Fig. 7a, c). By contrast, the positional isomer 2′,3′-cAMP was neither pro-apoptotic nor anti-proliferative (Fig. 7b, d). Adenosine, a potential metabolic product of both 2′,3′- or 3′,5′-cAMP, was inactive with regard to proliferation (Fig. 7d) but significantly active at 200 μM in the apoptosis assay (*p* < 0.01 compared with medium control; two-way ANOVA with Dunnet’s multiple comparison test; Fig. 7b). The cyclic pyrimidine nucleotides 3′,5′-cCMP and 3′,5′-cUMP that had been ineffective at inhibiting αCD3 antibody-induced IL2-release were also completely inactive with respect to apoptosis (Fig. 7a) and proliferation (Fig. 7c), even at a concentration of 200 μM. Likewise, the positional isomers 2′,3′-cCMP and 2′,3′-cUMP did not influence the readouts for apoptosis (Fig. 7b) and proliferation (Fig. 7d).

### Cytotoxicity of guanosine and guanosine-related compounds on PBMCs/T cells and acute lymphoblastic leukemia (ALL) xenografts

**Fig. 8:**
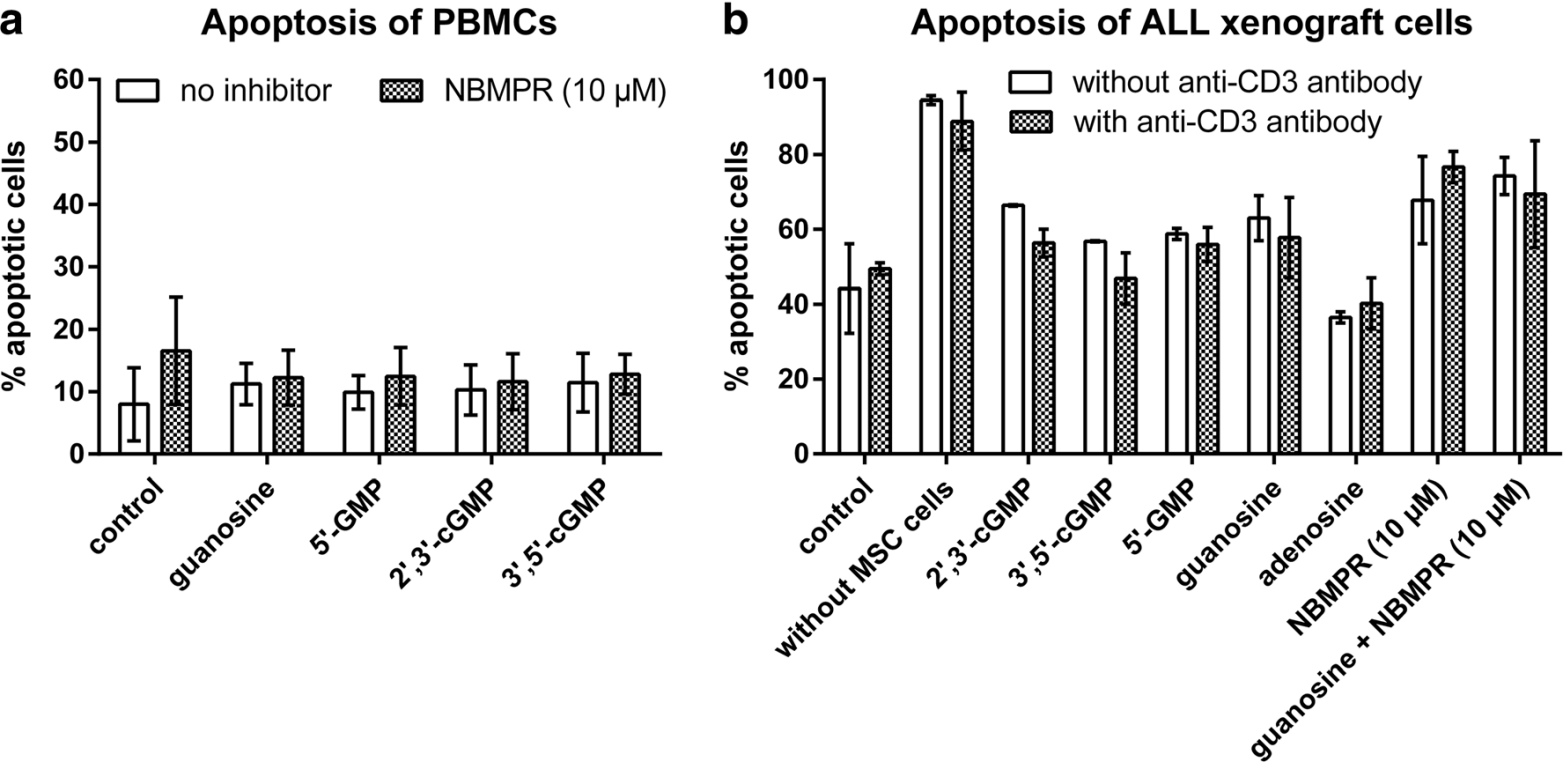
Apoptotic effects of guanosine-related compounds on PBMCs (**a**) and cortical T cell ALL xenograft cells (**b**). All compounds were tested at a concentration of 100 μM. PBMCs (**a**) were incubated for 72 h with 100 μM of the test compounds in the presence of anti-CD3- and anti-CD28-antibody. ALL xenograft cells (**b**) were co-cultured with mesenchymal stem cells (MSC cells) for 72 h in the absence of anti-CD3 antibody (open bars) and in the presence of anti-CD3 antibody (filled bars). The nucleoside transport inhibitor NBMPR was used at a concentration of 10 μM. Apoptosis was determined by combined staining with APC-labeled annexin V and propidium iodide. Data are means ± SD from *n* = 4 (**a**) or *n* = 2 (**b**) independent experiments

Since HuT-78 cells are lymphoma cells and the properties of cancer cell lines frequently differ from those of primary cells, we conducted additional experiments with freshly isolated PBMCs that also contain T lymphocytes. Since the PBMCs were incubated throughout the experiment (i.e., for 72 h) in the presence of anti-CD3 (OKT3) and anti-CD28 antibody, the proportion of T cells was further increased. In fact, these cells behaved very differently. Unlike in HuT-78 cells, none of the guanosine-related compounds induced apoptosis (Fig. 8a; open bars). Consequently, NBMPR (10 μM) did also not modulate any of these readouts in PBMCs (Fig. 8a; filled bars).

Since guanosine-related compounds were only active in HuT-78 cells but not in PBMCs, they may have therapeutic potential for the treatment of T cell lymphomas. Such compounds may selectively kill lymphoma cells without affecting the healthy T cell population. To elucidate whether the observed apoptotic and anti-proliferative effects also pertain to other lymphomas, we investigated the effect of 100 μM of 2′,3′-cGMP, 3′,5′-cGMP, 5′-GMP, and guanosine on cortical T cell acute lymphoblastic leukemia (ALL) xenograft cells. Adenosine (100 μM) was also investigated as a “non-guanosine negative control.” The xenografts had been previously produced by growing patient-derived tumor cells in immunologically deficient NSG mice (Frismantas et al. 2017). In contrast to conventional cancer cell lines, xenografts have the advantage that their characteristics are very close to primary cancer cells since they retain the pattern of mutations present in the original patient-derived material (Frismantas et al. 2017). Our experiment was conducted under similar conditions as used for the HuT-78 cells on anti-CD3-antibody-treated plates. However, unlike the HuT-78 cells, the xenograft cells were cultured in the presence of feeder cells (mesenchymal stem cells, MSC) as described by Frismantas et al. (2017). Moreover, a second set of wells was used to perform the experiment in the absence of anti-CD3 coating. Figure 8b shows that basal apoptosis under control conditions was already relatively high (40–50%) but independent of the presence of anti-CD3 antibody. Control wells without MSC feeder cells showed almost 100% apoptosis, indicating that MSC cells are absolutely necessary for the viability of the xenograft cells. The guanosine-derived compounds had no significant effect. Only a weak pro-apoptotic trend was observed (increase by about 10%, Fig. 8b). However, 10 μM of NBMPR did not protect the cells, when combined with 100 μM of guanosine (Fig. 8b). Interestingly, the lowest apoptosis rate was observed in the presence of 100 μM of adenosine, suggesting a protective effect.

## Discussion

### Apoptotic and anti-proliferative effects of guanosine-related compounds

**Fig. 9:**
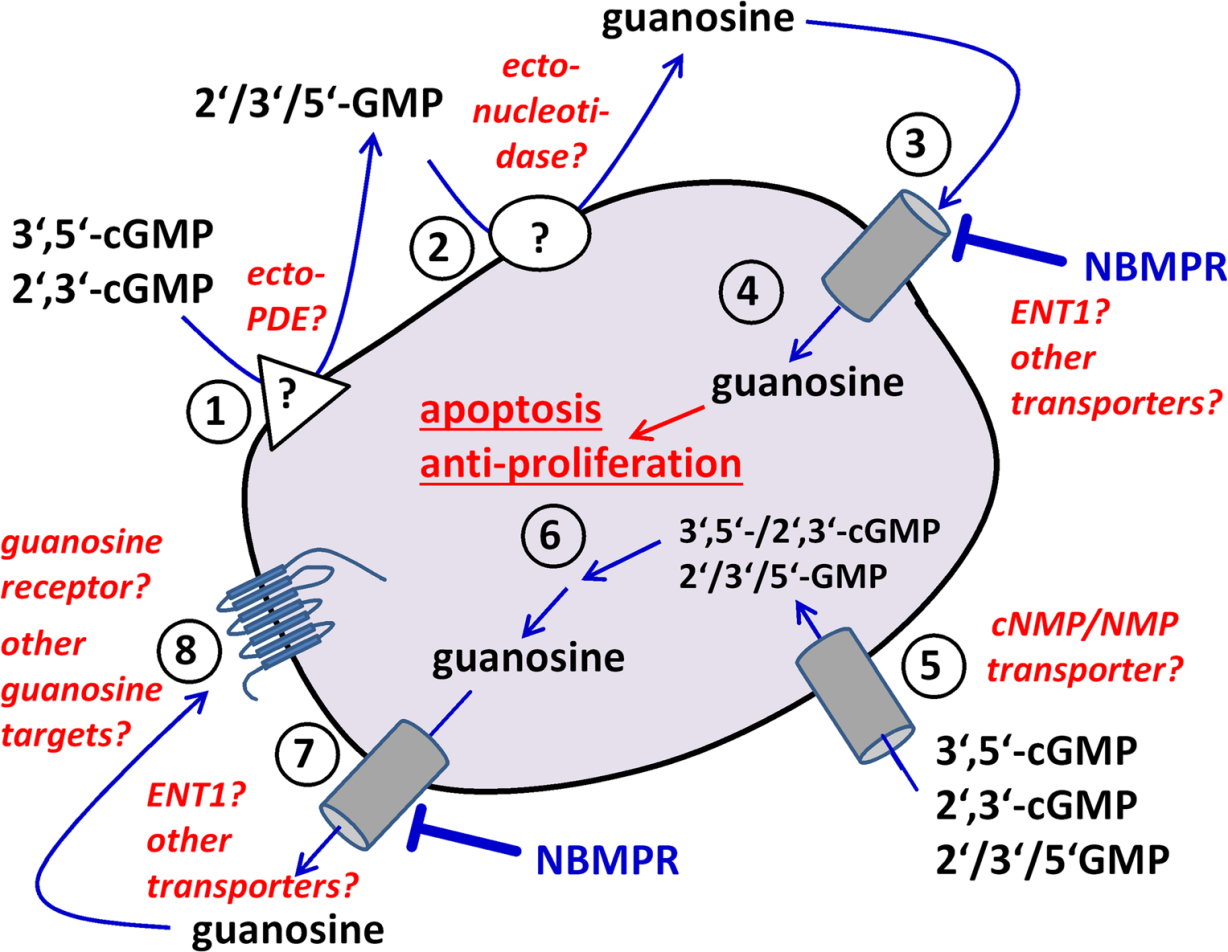
Hypotheses explaining the NBMPR-sensitive apoptotic and anti-proliferative effects of guanosine-derived compounds on HuT-78 cells. The numbers in the figure designate the following different factors/steps that might be involved: (1) 3′,5′-cGMP and 2′,3′-cGMP are hydrolyzed by a yet to be defined ecto-phosphodiesterase (ecto-PDE) on the cell surface. (2) The products of the ecto-PDE reaction, 5′-GMP (or 2′- or 3′-GMP), are metabolized by a still undefined ecto-nukleotidase, yielding guanosine. (3) Guanosine is taken up into the cell by an NBMPR-sensitive transporter (equilibrative nucleoside transporter ENT1?). (4) Intracellular guanosine causes the observed apoptotic and anti-proliferative effects. Alternative hypothesis, which is, however, considered less likely: (5) Uptake of 2′,3′−/3′,5′-cNMP and 5′-NMP (or 2′- or 3′-GMP) by yet to be defined membrane transporters. (6) Intracellular metabolism of 2′,3′−/3′,5′-cNMP and 5′-NMP (or 2′- or 3′-GMP) to yield guanosine. (7) Guanosine leaves the cell through an NBMPR-sensitive equilibrative nucleoside transporter (ENT1?) and (8) binds to a putative guanosine receptor (GPCR?) to cause apoptosis and growth inhibition

Prompted by the observation that 3′,5′-cGMP inhibits αCD3 antibody-stimulated IL-2 production of HuT-78 T-lymphoma cells, we have performed a detailed investigation of the effects of guanosine-related compounds on HuT-78 cell apoptosis and proliferation. We have mainly focused on apoptosis and proliferation data because they showed lower inter-experimental variability than anti-CD3 antibody-stimulated IL-2 production. The results presented in this publication demonstrate that guanosine as well as the guanosine-related nucleotides 2′,3′-cGMP, 3′,5′-cGMP, 2′-GMP, 3′-GMP, and 5′-GMP increase apoptosis and reduce proliferation of HuT-78 T lymphoma cells. It is unlikely that these effects are caused by activation of adenosine receptors because adenosine was active in apoptosis assays only at a high concentration of 200 μM and completely ineffective in proliferation experiments. Moreover, DPSPX, which is not only an ecto-phosphodiesterase inhibitor but also a non-selective adenosine receptor antagonist, did not eliminate the effects of the tested guanosine-related compounds. We developed several hypotheses to explain our observations (Fig. 9).

### First hypothesis: guanosine is formed extracellularly and taken up into HuT-78 cells by an NBMPR-sensitive nucleoside transporter

First, we hypothesized that the cytotoxic effects of 2′,3′−/3′,5′-cGMP as well as 2′-GMP, 3′-GMP and 5′-GMP are caused by guanosine, which is formed by enzymatic breakdown of the nucleotides and enters the cells through NBMPR-sensitive nucleoside transporters. The guanosine-cNMPs could be hydrolyzed to mononucleotides by ecto-phosphodiesterases (Fig. 9, step 1), followed by the formation of guanosine after further ectonucleotidase-mediated digestion (Fig. 9, step 2). In fact, metabolic pathways that lead to the formation of guanosine from cyclic guanine nucleotides have been described for 3′,5′-cGMP (Albrecht et al. 2013) and, very recently, for 2′,3′-cGMP (Jackson et al. 2019). A “2′,3′-cGMP-guanosine pathway” (Jackson et al. 2019) has been suggested in analogy to the previously described “2′,3′-cAMP-adenosine pathway” (Verrier et al. 2011; Jackson 2011).

Unexpectedly, the unselective ecto-phosphodiesterase inhibitor DPSPX did not eliminate the cytotoxic effects of 2′,3′- or 3′,5′-cGMP, and the ecto-5′-nucleotidase inhibitor AMP-CP did not counteract the apoptotic and anti-proliferative action of 5′-GMP. It is unlikely that these inhibitors were inactivated by hydrolysis during the 72 h incubation time because we did not even observe an effect when fresh AMP-CP or DPSPX were re-added after 24 h and 48 h. Thus, if guanosine was really formed from the guanosine-derived nucleotides in our experiments, the corresponding mechanism remains elusive.

The guanosine generated by guanosine nucleotide metabolism may be taken up by the cells through NBMPR-sensitive nucleoside transporters (Fig. 9, step 3) and then cause apoptotic and anti-proliferative effects by intracellular action (Fig. 9, step 4). It is well-known that extracellular nucleosides can be taken up by cells through specific transporters. As shown in Table 1, the various nucleoside transport processes can be divided in sodium-independent equilibrative (ENT) and sodium-dependent concentrative (CNT) processes. The ENT processes are bidirectional and comprise the *es* and the *ei* process. The hENT1 molecule is basically responsible for *es*, while the hENT2 transporter shows *ei* activity.

In our experiments with HuT-78 cells, 10 μM of NBMPR, an inhibitor of the human equilibrative nucleoside transporters hENT1 (IC_50_ = 0.4 nM) and hENT2 (IC_50_ = 2.8 μM), completely eliminated the cytotoxic effects of guanosine and guanosine-derived nucleotides. Additional experiments indicated that even 1 μM of NBMPR is already sufficient for the full protective effect. Concentration-effect curves with 100 μM of guanosine alone or in combination with increasing concentrations of NBMPR resulted in NBMPR IC_50_ values of 25 nM (apoptosis) and 28 nM (proliferation). The Cheng-Prusoff equation (Cheng and Prusoff 1973) (K_i_ = IC_50_/(1 + [S]/K_M_)) was employed with [S] being the concentration of the substrate guanosine (100 μM) and K_M_ representing the guanosine K_M_ value. Using the K_M_ value of guanosine for hENT1 for the calculation (140 μM, Table 1) yielded NBMPR K_i_ values of ~ 14.6 nM (apoptosis) and of 16.3 nM (proliferation). This is still ~ 40-fold higher than the literature NBMPR K_d_ (high-affinity [^3^H]NBMPR binding) at hENT1 (0.38 nM; Ward et al. 2000), which may be due to the fact that we did not determine the direct effect of NBMPR on guanosine transporter activity but used an indirect downstream parameter (apoptosis or proliferation). By contrast, an alternative calculation using the guanosine affinity for hENT2 (2700 μM, Table 1) resulted in a K_i_ of 24.1 nM (apoptosis assays) or 27 nM (proliferation experiments), which is more than 100-fold lower than the NBMPR IC_50_ described for hENT2 in the literature (2.8 µM; Ward et al. 2000). Unfortunately, no NBMPR K_d_ value was reported by Ward et al (2000) for hENT2.

In summary, our results suggest involvement of hENT1 rather than hENT2 in producing the cytotoxic effects of guanosine. It should be noted, however, that NBMPR does not only inhibit ENT1 but also the concentrative *csg* transport process that also accepts guanosine. The *csg* process (Table 1) was first functionally characterized in NB4 acute promyelocytic leukemia cells (Flanagan and Meckling-Gill 1997). Thus, our experiments currently cannot differentiate between ENT1 (*es*) and *csg* in HuT-78 cells. Future experiments should therefore strive for detecting the presence of hENT1 on the protein level in HuT-78 cells. By contrast, expression of the transporter for the *csg* process cannot be investigated because, to the best of our knowledge, its molecular identity is still elusive.

As far as we know, relevant transport of 2′,3′-cGMP, 3′,5′-cGMP, 2′-GMP, 3′-GMP, or 5′-GMP by the NBMPR-sensitive transport processes *es* or *csg* has not been reported so far. Thus, the cytoprotective effect of NBMPR in our experiments supports the notion that guanosine is formed as common end-product of guanosine nucleotide metabolism and is in fact the active principle after intracellular uptake by guanosine transporters.

### Second hypothesis: guanosine is formed intracellularly and exported by an NBMPR-sensitive nucleoside transporter

Alternatively, according to our second hypothesis, 2′,3′-, 3′,5′-cGMP, 2′-GMP, 3′-GMP and 5′-GMP could enter the cell via yet to be identified transporters (Fig. 9, step 5). For example, uptake of 3′,5′-cGMP could be facilitated by the organic anion transporter OAT2 and, to a minor extent, by OAT1 or 3 (Henjakovic et al. 2015). The spectrum of guanine nucleotides transported by OAT2 seems to be rather broad as it also accepts 2′-deoxyguanosine, GMP, GDP, and GTP (Cropp et al. 2008). Guanosine may then be formed *intracellularly* (Fig. 9, step 6) and leave the cell through a (bidirectional!) NBMPR-sensitive transporter, possibly hENT1 (Fig. 9, step 7). After that, guanosine may cause apoptosis by *extracellular* action, e.g. via putative guanosine receptors or other yet to be defined target sites (Fig. 9, step 8). Not much is known about guanosine receptors. Several years ago, a G protein-coupled guanosine receptor has been postulated in the rat brain on the basis of data from [^3^H]guanosine radioligand binding assays (Traversa et al. 2003, 2002), cAMP accumulation assays (Traversa et al. 2003), or europium-based Gα-activation assays (Volpini et al. 2011). However, to the best of our knowledge, the molecular identity of this binding site is still elusive.

Since OATs are largely NBMPR insensitive, the second hypothesis would explain the inhibitory effect of NBMPR by a reduction of guanosine export from the cells. However, since this hypothesis assumes *extracellular* action of guanosine, it cannot explain why extracellularly applied guanosine acts in an NBMPR-sensitive way. Perhaps reality comprises a mixture of mechanisms from the first and the second hypothesis.

### Different effects of cGMP and cGMP-AM on IL-2 release and apoptosis/proliferation

The ELISA data in Fig. 1 show that cGMP-AM does not affect IL-2 release, while 3′,5′-cGMP has a pronounced inhibitory effect. By contrast, a weak and non-significant inhibitory effect of cGMP-AM on IL-2 release was visible in later experiments (Suppl. Fig. 3). It is noted, however, that the data in Suppl. Fig. 3 show large variability and the IL-2 release is only very low and not influenced by αCD3. Thus, the results regarding the cGMP-AM effect on IL-2 production are rather inconclusive in Suppl. Fig. 3. By contrast, cGMP-AM had a clear pro-apoptotic and anti-proliferative effect (Suppl. Fig. 2), which was comparable with the cytotoxic effects of the other tested guanosine-related compounds.

We assume that the difference in cGMP-AM effect between IL2 ELISAs and apoptosis/proliferation experiments is due to the longer incubation time of 72 h in the apoptosis/proliferation assays as compared to only 24 h in the ELISA experiments. Previous pilot experiments (data not shown) indicated that it took 72 h for 5′-GMP and guanosine to produce pronounced effects on apoptosis and proliferation. By contrast, IL-2 production by HuT-78 cells may follow a different time course. Moreover, intracellular delivery of cGMP from cGMP-AM may be more important during the first 24 h of incubation. By contrast, during longer incubation times, hydrolysis of cGMP-AM in the medium may produce large amounts of extracellular cGMP, which makes it difficult to discriminate between intracellular and extracellular actions of cGMP derived from cGMP-AM. The difference between the effects of cGMP and cGMP-AM and the underlying mechanisms should be investigated in more detail in future studies.

### (Patho)physiological roles of extracellular cGMP

In our experiments, we have added extracellular cGMP to HuT-78 cell cultures. At first glance, this seems to be an odd way to investigate cGMP effects, as cGMP is widely recognized as an intracellular second messenger. However, (patho)physiological roles of extracellular cGMP have been demonstrated in the past. Suppl. Table 1 shows several examples of extracellular cGMP effects reported in the literature. Extracellular cGMP modulates natriuresis in the kidneys. It seems to interfere with the effect of various platelet-activating agents. Moreover, extracellular cGMP appears to act in the gastrointestinal tract, e.g. by modulating fluid absorption and secretion as well as visceral sensitivity. Numerous effects of extracellular cGMP have been reported in the CNS, mainly in animal models of hepatic hyperammonemic encephalopathy. The references in Suppl. Table 2 suggest that the effect of extracellular cGMP seems at least partly mediated by conversion to guanosine. However, Suppl. Tables 1 and 2 show mostly neuroprotective effects of extracellular cGMP and guanosine, while our results rather support apoptotic and anti-proliferative action of extracellular cGMP in HuT-78 cells. This difference suggests that extracellular cGMP exerts very distinct cell type-dependent effects.

### Nucleoside transporters and nucleoside-derived cytostatic drugs

Nucleoside transporters are an important prerequisite for the activity of nucleoside-derived drugs, e.g., several anti-cancer agents. The NBMPR-sensitive transporter ENT1 mediates uptake of the cytostatic drugs gemcitabine, fludarabine, cladribine, and cytarabine (Table 1) (Pastor-Anglada et al. 2004). CEM lymphoblastic leukemia cells express ENT1 and are sensitive to gemcitabine (Mackey et al. 1998). However, in the presence of NBMPR, the IC_50_ of gemcitabine increased by more than a 100-fold (Mackey et al. 1998). Moreover, it has been recently reported that the imidazole nucleoside immunosuppressant mizoribine is taken up by L5178Y-R mouse lymphoma cells and metabolized to the corresponding monophosphate. The L5178Y-R cells express mRNA for both ENT1 and ENT2. Mizoribine uptake was inhibited by the unselective ENT1/ENT2 substrate adenosine but not by 0.1 μM of the ENT1 inhibitor NBMPR (Oda et al. 2018).

Our results suggest that guanosine and/or guanosine-related compounds could be an important addition to the cytostatic therapy of specific kinds of leukemia. The efficacy, however, may be highly dependent on the presence of the corresponding nucleoside transporters, e.g. ENT1 or *csg*. This has been demonstrated for several established cytostatic drugs. For example, CEM-ARA-C/8C cells are virtually resistant to gemcitabine because they are deficient of any kind of nucleoside transport (Mackey et al. 1998). Later, it has been reported that CEM-ARA-C/8C cells still express ENT1, but a glycine-to-arginine mutation in the ENT1 protein (G24R) results in a loss of nucleoside uptake activity (Zimmerman et al. 2009). Moreover, it has been proposed that immunohistochemical determination of hENT1 expression may be useful to predict gemcitabine or capecitabine resistance of breast cancer cells (Mackey et al. 2002).

Lack of nucleoside transporter expression in some cancers may cause resistance to nucleoside-derived cytostatic drugs. This may be the reason, why guanosine and guanosine-related compounds were practically ineffective in our experiments with ALL xenograft cells. In fact, it has been reported that *es* transporter expression in fresh leukemic lymphoblasts isolated from four different ALL patients showed considerable inter-individual variability that was reflected by very heterogeneous cytostatic effects of cytarabine (Gati et al. 1998). It should be noted, however, that no information about the exact type of ALL was provided by Gati et al. (1998). We hypothesize that the ALL clone used in our experiments may have shown a rather low expression of guanosine-transporting proteins. Thus, as previously recommended for breast cancer (Mackey et al. 2002), also in case of different kinds of leukemia, a pre-screening for nucleoside transporter expression may help to select patients responsive to therapy with nucleoside-derived cytostatics. In this regard, it should be noted that there may be other, NBMPR-insensitive, transport processes that are able to mediate guanosine uptake. For example, guanosine increased apoptosis of Jurkat (human T cell leukemia) cells in an NBMPR-insensitive way (Batiuk et al. 2001).

### Nucleoside transporters in PBMCs

We can only speculate why guanine nucleotides and guanosine did not affect viability and proliferation of PBMCs in our experiments. Since we have cultured the PBMCs during our experiments for 72 h in the presence of αCD3- and αCD28 antibody, we assume that proliferation of T cells was selectively stimulated and T cells were enriched in our culture. Very recently, it has been demonstrated that peripheral T cells show very high abundance of mRNA for ENT3 but only very low amounts of ENT1 or ENT2 mRNA (Wei et al. 2018). ENT3, however, is mainly expressed intracellularly in lysosomal and mitochondrial membranes (Govindarajan et al. 2009) and therefore unable to mediate uptake of extracellular nucleosides. By contrast, another publication reports hENT1 mRNA in PBMCs, but experiments with tritiated [^3^H]gemcitabine revealed that the concentrative nucleoside transporter hCNT1 contributed much more to [^3^H]gemcitabine uptake than hENT1 (Choi 2012). To the best of our knowledge, hCNT1 mainly accepts pyrimidine nucleosides as substrates and no guanosine transport by hCNT1 has been reported till now (Pastor-Anglada et al. 2004). Alternatively, PBMCs could take up guanosine by other transporters (e.g. hCNT2 or hCNT3) but may be resistant to the apoptotic effects of guanosine.

### Conclusion and outlook

We have identified apoptotic and anti-proliferative effects of guanosine and guanosine-related compounds in HuT78 human Sézary lymphoma cells. These effects seem to strongly depend on the activity of an NBMPR-sensitive nucleoside transporter, the identity of which is still elusive. Our concentration effect curves with NBMPR suggest that ENT2 is most likely not involved. We propose that guanosine and related compounds could serve as potential adjuvants for the treatment of various kinds of guanosine transporter-positive cancers, in addition to established cytostatic nucleoside analogues.

Future studies will have to clarify if HuT-78 cells take up guanosine via the *es* (ENT1) and/or the *csg* process. Moreover, other cancer cell lines should be tested for their guanosine sensitivity, and the data should be correlated with expression levels of ENT1 (e.g. as determined by [^3^H]NBMPR binding) or with the guanosine uptake capacity (e.g. uptake of [^3^H]guanosine). Moreover, it should be investigated whether extracellular effects of guanosine play a role in some cell lines, e.g. cytotoxicity mediated by adenosine receptors (Oliveira et al. 2017). Finally, the effect of guanosine should be investigated in mouse leukemia or lymphoma models since the guanosine-accepting *es* nucleoside transport process (probably caused by ENT1) was detected in murine lymphoma (S49) and leukemia (L1210) cancer cell lines (Mackey et al. 1998).

## Electronic supplementary material

ESM 1 (PDF 666 kb).

## Abbreviations

*ALL*: Acute lymphocytic leukemia
*AMP-CP*: Adenosine-5′-(α,β-methylene)diphosphate, a CD73 ectonucleotidase inhibitor
*2′,3′-* or *3′,5′-cAMP*: Adenosine 2′,3′- or 3′,5′-cyclic monophosphate
*Annexin V-APC*: Allophycocyanin-labeled annexin V
*BSA*: Bovine serum albumin
*cCMP*: Cytidine 3′,5′-cyclic monophosphate
*CEM-ARA-C/8C*: Arabinosylcytosine-resistant childhood T acute lymphoblastic leukemia cell line
*CFSE*: 5(6)-Carboxyfluorescein diacetate N-succinimidyl ester
*2′,3′-* or *3′,5* ′*-cGMP*: Guanosine 2′,3′- or 3′,5′-cyclic monophosphate
*cIMP*: Inosine 3′,5′-cyclic monophosphate
*cNMP*: Cyclic nucleoside monophosphate
*cNMP-AM*: Cyclic nucleoside 3′,5′ monophosphate acetoxymethyl ester
*cUMP*: Uridine 3′,5′-cyclic monophosphate
*DPSPX*: 1,3-Dipropyl-8-p-sulfophenylxanthine, an ecto-phosphodiesterase inhibitor
*EDTA*: Ethylenediaminetetraacetic acid
*ELISA*: Enzyme-linked immunosorbent assay
*3′- or 5′-GMP*: Guanosine 3′- or 5′-monophosphate
*GPCR*: G protein-coupled receptor
*hCNT1–3*: Human concentrative nucleoside transporters, isoforms 1-3
*hENT1–4*: Human equilibrative nucleoside transporters, isoforms 1-4
*HuT-78 cells*: Human T cell lymphoma (Sézary lymphoma) cell line
*IL-2*: Interleukin 2
*MSC*: Mesenchymal stem cells
*NB4 cells*: Human acute promyelocytic leukemia
*NBMPR*: 6-S-[(4-Nitrophenyl)methyl]-6-thioinosine, an inhibitor of the equilibrative nucleoside transporter ENT1
*NMP*: Nucleoside monophosphate
*OAT*: Organic anion transporter
*PBMCs*: Peripheral blood mononuclear cells
*PBS*: Phosphate buffered saline
*PDE*: Phosphodiesterase
*PI*: Propidium iodide
*PMAT*: Plasma membrane monoamine transporter
*PO*_*4*_*(AM)*_*3*_: Phosphate tris(acetoxymethyl)-ester (control compound for cNMP-AMs)
*SD*: Standard deviation
*TCR*: T cell receptor
