# Supplementary material for: Apoptotic and anti-proliferative effect of guanosine and guanosine derivatives in HuT-78 T lymphoma cells
Source: Naunyn Schmiedebergs Arch Pharmacol. 2020 Apr 20;393(7):1251–67. doi: 10.1007/s00210-020-01864-8 (PMC7314729; doi:10.1007/s00210-020-01864-8)
Supplement: Supplementary file 1 — (PDF 666 kb). [file 210_2020_1864_MOESM1_ESM.pdf]

Supplemental Tables and Figures

Supplemental Table 1: Publications that suggest physiological effects of extracellular cGMP. Please note that this table is not exhaustive and only comprises selected examples of literature about extracellular cGMP actions.

**Renal effects of extracellular cGMP**	
ANP stimulation of the basal pole of filter-grown human podocyte HGVEC.SV1A4 cells leads to release of cGMP into the apical medium.	(Ardaillou et al., 1992)
SNP inhibits sodium transport through porcine renal tubular LLC-PK1 cell monolayers, which depends on probenecid-sensitive cGMP extrusion.	(Chevalier et al., 1996)
NO stimulates cGMP generation and extrusion by human renal proximal tubule cells. Extracellular cGMP reduces cellular sodium uptake.	(Sasaki et al., 2004)
Extracellular cGMP (exported from its renal synthesizing cells) causes the natriuretic effect of the NO donor SNAP.	(Ahmed et al., 2007)
Src is an important downstream signaling molecule for extracellular cGMP-induced natriuresis.	(Nascimento et al., 2011)
**Gastrointestinal effects of extracellular cGMP**	
cGMP in the serosal part of the jejunum increases fluid absorption, but mucosal activation by heat-stable enterotoxin from *E. coli* increases loop cGMP and fluid secretion.	(Jin et al., 1999)
Tapeworms release cGMP at a constant rate to the intestinal lumen. [^{3}H]cGMP radioligand binding data suggest mucosal cGMP binding sites for extracellular cGMP.	(Zimmerman et al., 2008)
Uroguanylin is antihyperalgesic in a model of trinitrobenzene sulfonic acid (TNBS)-induced visceral hypersensitivity. Oral cGMP mimicks the antihyperalgesic effects of uroguanylin.	(Silos-Santiago et al., 2013)
The hardly absorbed guanylate cyclase-C agonist linaclotide reduces symptoms of irritable bowel syndrome with constipation. In the mouse model, this effect appears to be mediated by extracellular cGMP.	(Castro et al., 2013)
**Effects of extracellular cGMP on platelet activation**	
Extracellular cGMP analogs potently and rapidly inhibit thrombin-, thromboxane-, and VWF-induced human platelet signaling and activation by a cGK-independent mechanism.	(Gambaryan et al., 2004)
**Effects of extracellular cGMP in the CNS**	
Extracellular cGMP reduces intracellular pH in cultured rat astrocytes by inhibiting a novel type of astrocytic Na^+/H^+ exchanger.	(Touyz et al., 1997)
Extracellular cGMP directly inhibits kainate-activated responses in a subpopulation of cultured mouse cerebellar neurons.	(Poulopoulou and Nowak, 1998)
Intracellular cGMP is neurotoxic, but extracellular cGMP protects from glutamate neurotoxicity in cultured primary cerebellar neurons.	(Montoliu et al., 1999)
Rats with hyperammonemia show impaired learning ability and impaired function of the brain glutamate-NO-cGMP pathway. Oral administration of the phosphodiesterase 5 inhibitor sildenafil increases extracellular cGMP and restores learning ability in a conditional discrimination task.	(Erceg et al., 2005)

Learning ability was impaired in hyperammonemic rats, but restored by cGMP PDE inhibitors or continuous intracerebral cGMP administration.	(Rodrigo et al., 2006) [Review]
The neurotransmitter release evoked by activation of presynaptic kainate receptors is inhibited by extracellular cGMP. The AMPA (α-amino-3-hydroxy-5-methyl-4-isoxazolepropionic acid) autoreceptor-mediated response, however, is not affected by cGMP.	(Cervetto et al., 2010)
Administration of extracellular cGMP normalizes spatial reference memory (probably by modulation of TNF-α and of membrane expression of AMPA receptor GluA1 and GluA2 subunits), but does not normalize impairment of working memory in hyperammonemic rats.	(Cabrera-Pastor et al., 2016a)
Extracellular cGMP reduces glycine receptor activation, which increases intracellular Ca^{2+} in Purkinje neurons via voltage-dependent Ca^{2+}-channels. Extracellular cGMP increases learning ability, when basal calcium concentration is low, but reduces learning ability, when basal calcium is normal.	(Cabrera-Pastor et al., 2016b)
Extracellular cGMP reduces glycine receptor activation and modulates membrane expression of AMPA receptors by increasing GluA1 and reducing GluA2 subunit expression.	(Cabrera-Pastor et al., 2017)
Chronic intracerebral administration of extracellular cGMP restores motor coordination in hyperammonemic rats by reducing microglia activation and neuroinflammation. Extracellular cerebellar glutamate and GABA levels are normalized by cGMP administration.	(Cabrera-Pastor et al., 2018)
Increasing extracellular cGMP normalizes expression of various transporters for glutamate, glutamine and GABA in the cerebellum of rats with chronic hyperammonemia.	(Cabrera-Pastor et al., 2019)

Supplemental Table 2: Physiological effects by guanosine formed as a metabolite of GMP or cGMP. Please note that this table is not exhaustive and only comprises selected examples of literature about actions of extracellular guanosine generated by GMP or cGMP metabolism.

Effect	Reference
GMP exerts an anticonvulsant effect that appears to depend on its conversion to guanosine by ecto-5'-nucleotidase.	(Soares et al., 2004)
The amnesic effect of GMP is prevented by inhibiting its conversion to guanosine. This conversion occurs not only in the CNS, but also in the periphery.	(Saute et al., 2006)
In neuronal HT22 cells, extracellular glutamate causes glutathione depletion, ROS (reactive oxygen species) elevation and apoptosis (by cytotoxic Ca^{2+} influx). Extracellular cGMP as well as GMP was protective. The protective effect of extracellular GMP depended on its conversion to guanosine.	(Albrecht et al., 2013)

Suppl. Fig. 1

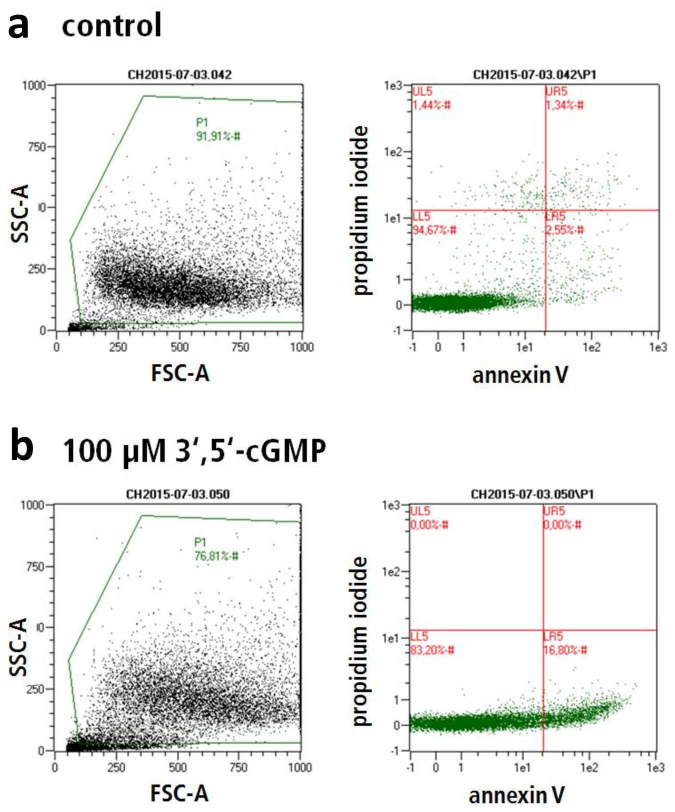

Suppl. Fig. 1: Quantification of 3',5'-cGMP-induced apoptosis of HuT-78 cells by flow cytometry (staining with propidium iodide + annexin V), representative measurement. (**a**) control; (**b**) after incubation with 100 µM of 3',5'-cGMP for 72 h; left side: gating of the cells in the FSC/SSC scattergram; right side: distribution with regard to propidium iodide and annexin V staining. The percentage of cells in the lower right (LR) and upper right (UR) quadrant represents the percentage of apoptotic cells.

Suppl. Fig. 2

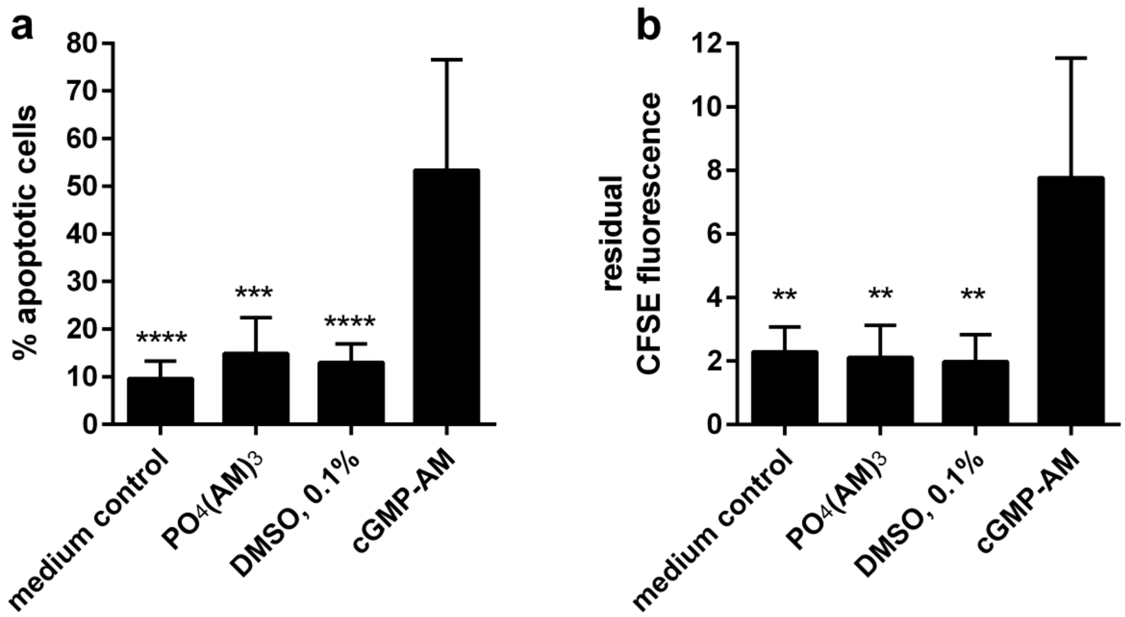

Suppl. Fig. 2: Effect of 100 µM of cGMP-AM on apoptosis (**a**) and proliferation (**b**) in comparison to medium control, $PO_4(AM)_3$ and DMSO 0.1% as negative controls. Apoptosis **(a)** and proliferation **(b)** were determined by flow cytometry after 72 h of incubation (apoptosis: combined staining with APC-labeled annexin V and propidium iodide; proliferation: quantitation of residual CFSE fluorescence at the end of the incubation time, higher residual CFSE fluorescence means less proliferation). Data are means ± SD from n=6 (apoptosis) or n=5 (proliferation) independent experiments. Statistics: One way ANOVA and Dunnet's multiple comparison test, with "*" indicating significance in comparison to cGMP-AM; two, three and four symbols designate $p<0.01$, $p<0.001$ and $p<0.0001$, respectively.

Suppl. Fig. 3

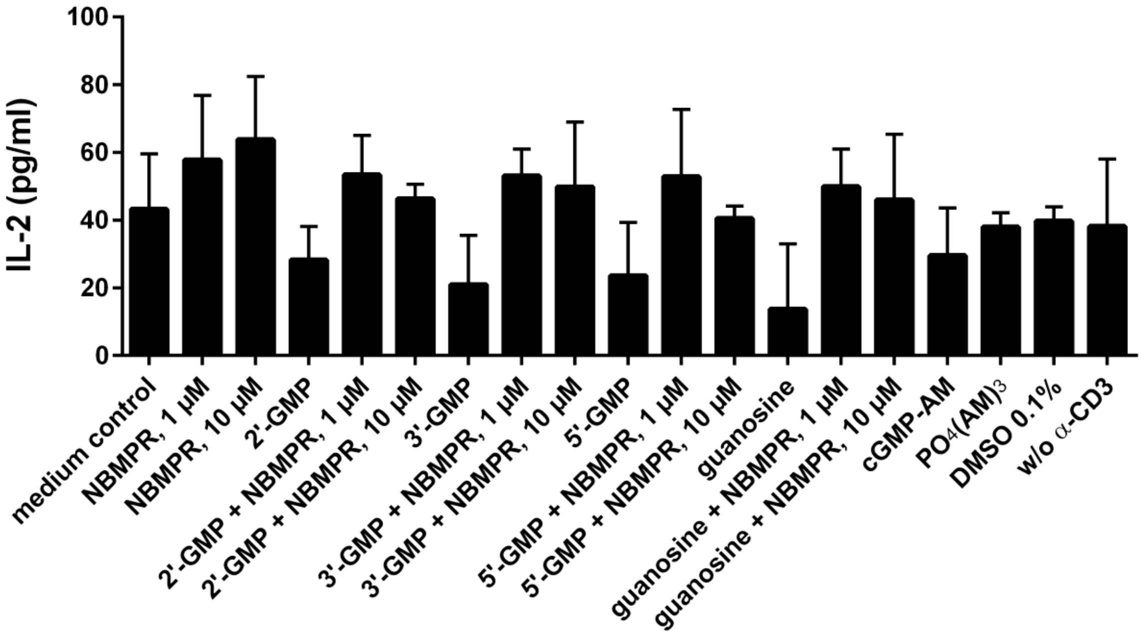

Suppl. Fig. 3: Effect of potential products of 2',3'-/3',5'-cGMP metabolism (100 µM of 2'-GMP, 3'-GMP, 5'-GMP, guanosine) on IL-2 production of HuT-78 lymphoma cells in the absence and in the presence of NBMPR. cGMP-AM (100 µM) was included as a control for intracellularly delivered cGMP. Cells were incubated for 24 h in αCD3-antibody-coated plates with 100 µM of the corresponding guanosine-derived compounds in the absence and in the presence of NBMPR (1 µM and 10 µM). The "DMSO 0.1%" sample is a solvent control for the cGMP-AM sample. The "$PO_4(AM)_3$" is another control for the cGMP-AM sample, which imitates the intracellular formation of acetoxymethylester hydrolysis products. For unknown reasons, the HuT-78 cells only showed a low basal IL-2 production in these experiments that was not further enhanced by αCD3-antibody. Data are means ± SD from n=3 independent experiments. Statistics: One way ANOVA and Dunnet's multiple comparison test with medium control as control column (no significant difference detected).

References

Ahmed F, Kemp BA, Howell NL, Siragy HM, Carey RM (2007) Extracellular renal guanosine cyclic 3'5'-monophosphate modulates nitric oxide and pressure-induced natriuresis. Hypertension 50: 958-963

Albrecht P, Henke N, Tien ML, Issberner A, Bouchachia I, Maher P, Lewerenz J, Methner A (2013) Extracellular cyclic GMP and its derivatives GMP and guanosine protect from oxidative glutamate toxicity. Neurochem Int 62: 610-619

Ardaillou N, Lelongt B, Turner N, Piedagnel R, Baudouin B, Estrade S, Cassingena R, Ronco PM (1992) Characterization of a simian virus 40-transformed human podocyte cell line producing type IV collagen and exhibiting polarized response to atrial natriuretic peptide. J Cell Physiol 152: 599-616

Cabrera-Pastor A, Arenas YM, Taoro-Gonzalez L, Montoliu C, Felipo V (2019) Chronic hyperammonemia alters extracellular glutamate, glutamine and GABA and membrane expression of their transporters in rat cerebellum. Modulation by extracellular cGMP. Neuropharmacology, doi: 10.1016/j.neuropharm.2019.01.011. [Epub ahead of print]

Cabrera-Pastor A, Balzano T, Hernández-Rabaza V, Malaguarnera M, Llansola M, Felipo V (2018) Increasing extracellular cGMP in cerebellum in vivo reduces neuroinflammation, GABAergic tone and motor in-coordination in hyperammonemic rats. Brain Behav Immun 69: 386-398

Cabrera-Pastor A, Hernandez-Rabaza V, Taoro-Gonzalez L, Balzano T, Llansola M, Felipo V (2016a) In vivo administration of extracellular cGMP normalizes TNF-α and membrane expression of AMPA receptors in hippocampus and spatial reference memory but not IL-1β, NMDA receptors in membrane and working memory in hyperammonemic rats. Brain Behav Immun 57: 360-370

Cabrera-Pastor A, Malaguarnera M, Taoro-Gonzalez L, Llansola M, Felipo V (2016b) Extracellular cGMP Modulates Learning Biphasically by Modulating Glycine Receptors, CaMKII and Glutamate-Nitric Oxide-cGMP Pathway. Sci Rep 6: 33124

Cabrera-Pastor A, Taoro-González L, Cuñat AN, Canet-López D, Balzano T, Felipo, V (2017) Extracellular Cyclic GMP Modulates Membrane Expression of The GluA1 and GluA2 Subunits of AMPA Receptor in Cerebellum: Molecular Mechanisms Involved. Sci Rep 7: 17656

Castro J, Harrington AM, Hughes PA, Martin CM, Ge P, Shea CM, Jin H, Jacobson S, Hannig G, Mann E, Cohen MB, MacDougall JE, Lavins BJ, Kurtz CB, Silos-Santiago I, Johnston JM, Currie MG, Blackshaw LA, Brierley SM (2013) Linaclotide inhibits colonic nociceptors and relieves abdominal pain via guanylate cyclase-C and extracellular cyclic guanosine 3',5'-monophosphate. Gastroenterology 145: 1334-1346.e1-11.

Cervetto C, Maura G, Marcoli M (2010) Inhibition of presynaptic release-facilitatory kainate autoreceptors by extracellular cyclic GMP. J Pharmacol Exp Ther 332: 210-219

Chevalier RL, Fang GD, Garmey M (1996) Extracellular cGMP inhibits transepithelial sodium transport by LLC-PK1 renal tubular cells. Am J Physiol 270: F283-288

Erceg S, Monfort P, Hernández-Viadel M, Rodrigo R, Montoliu C, Felipo V (2005) Oral administration of sildenafil restores learning ability in rats with hyperammonemia and with portacaval shunts. Hepatology 41: 299-306

Gambaryan S, Geiger J, Schwarz UR, Butt E, Begonja A, Obergfell A, Walter U (2004) Potent inhibition of human platelets by cGMP analogs independent of cGMP-dependent protein kinase. Blood 103: 2593-2600

Jin XH, Siragy HM, Guerrant RL, Carey RM (1999) Compartmentalization of extracellular cGMP determines absorptive or secretory responses in the rat jejunum. J Clin Invest 103: 167-174

Montoliu C, Llansola M, Kosenko E, Corbalán R, Felipo V (1999) Role of cyclic GMP in glutamate neurotoxicity in primary cultures of cerebellar neurons. Neuropharmacology 38: 1883-1891

Nascimento NR, Kemp BA, Howell NL, Gildea JJ, Santos CF, Harris TE, Carey RM (2011) Role of SRC family kinase in extracellular renal cyclic guanosine 3',5'-monophosphate- and pressure-induced natriuresis. Hypertension, 58: 107-113

Poulopoulou C, Nowak LM (1998) Extracellular 3',5' cyclic guanosine monophosphate inhibits kainate-activated responses in cultured mouse cerebellar neurons. J Pharmacol Exp Ther, 286: 99-109

Rodrigo R, Monfort P, Cauli O, Erceg S, Felipo V (2006) Pharmacological manipulation of cyclic GMP levels in brain restores learning ability in animal models of hepatic encephalopathy: therapeutic implications. Neuropsychiatr Dis Treat 2: 53-63

Sasaki S, Siragy HM, Gildea JJ, Felder RA, Carey RM (2004) Production and role of extracellular guanosine cyclic 3', 5' monophosphate in sodium uptake in human proximal tubule cells. Hypertension 43: 286-291

Saute JA, da Silveira LE, Soares FA, Martini LH, Souza DO, Ganzella M (2006) Amnesic effect of GMP depends on its conversion to guanosine. Neurobiol Learn Mem 85: 206-212

Silos-Santiago I, Hannig G, Eutamene H, Ustinova EE, Bernier SG, Ge P, Graul C, Jacobson S, Jin H, Liong E, Kessler MM, Reza T, Rivers S, Shea C, Tchernychev B, Bryant AP, Kurtz CB, Bueno L, Pezzone MA, Currie MG (2013) Gastrointestinal pain: unraveling a novel endogenous pathway through uroguanylin/guanylate cyclase-C/cGMP activation. Pain 154: 1820-1830

Soares FA, Schmidt AP, Farina M, Frizzo ME, Tavares RG, Portela LV, Lara DR, Souza DO (2004) Anticonvulsant effect of GMP depends on its conversion to guanosine. Brain Res 1005: 182-186

Touyz RM, Picard S, Schiffrin EL, Deschepper CF (1997) Cyclic GMP inhibits a pharmacologically distinct Na^+/H^+ exchanger variant in cultured rat astrocytes via an extracellular site of action. J Neurochem 68: 1451-1461

Zimmerman NP, Brownfield MS, DeVente J, Bass P, Oaks JA (2008) cGMP secreted from the tapeworm Hymenolepis diminuta is a signal molecule to the host intestine. J Parasitol 94: 771-779
